# New Polyesterified Ursane Derivatives from Leaves of *Maesa membranacea* and Their Cytotoxic Activity

**DOI:** 10.3390/molecules26227013

**Published:** 2021-11-20

**Authors:** Klaudia Michalska, Agnieszka Galanty, Thanh Nguyen Le, Janusz Malarz, Nguyen Quoc Vuong, Van Cuong Pham, Anna Stojakowska

**Affiliations:** 1 Maj Institute of Pharmacology, Polish Academy of Sciences, Smętna Street 12, 31-343 Kraków, Poland; klaudiaz@if-pan.krakow.pl (K.M.); malarzj@if-pan.krakow.pl (J.M.); 2 Department of Pharmacognosy, Collegium Medicum, Jagiellonian University, Medyczna Street 9, 30-688 Kraków, Poland; mfgalant@cyf-kr.edu.pl; 3 Institute of Marine Biochemistry, Graduate University of Science and Technology, Vietnam Academy of Science and Technology, 18 Hoang Quoc Viet, Caugiay, Hanoi 1000000, Vietnam; lethanh@imbc.vast.vn (T.N.L.); nguyenvuong@imbc.vast.vn (N.Q.V.); phamvc@imbc.vast.vn (V.C.P.)

**Keywords:** cytotoxicity, *Maesa membranacea*, Maesaceae, Primulaceae, prostate cancer, triterpenoids, ursane

## Abstract

*Maesa membranacea* A. DC. (Primulaceae) is a plant species that has been frequently used by practitioners of the traditional ethnobotany knowledge from northern and central Vietnam. However, the chemical constituents of the plant remained unknown until recently. Chromatographic separation of a chloroform-soluble fraction of extract from leaves of *M. membranacea* led to the isolation of two new polyesterified ursane triterpenes (**1**–**2**) and two known apocarotenoids: (+)-dehydrovomifoliol (**3**) and (+)-vomifoliol (**4**). The chemical structures of the undescribed triterpenoids were elucidated using 1D and 2D MNR and HRESIMS spectral data as 2*α*,6*β*,22*α*-triacetoxy-11*α*-(2-methylbutyryloxy)-urs-12-ene-3*α*,20*β*-diol (**1**) and 2*α*,6*β*,22*α*-triacetoxy-urs-12-ene-3*α*,11*α*,20*β*-triol (**2**). The newly isolated triterpenoids were tested for their cytotoxic activity in vitro against two melanoma cell lines (HTB140 and A375), normal skin keratinocytes (HaCaT), two colon cancer cell lines (HT29 and Caco-2), two prostate cancer cell lines (DU145 and PC3) and normal prostate epithelial cells (PNT-2). Doxorubicin was used as a reference cytostatic drug. The 2*α*,6*β*,22*α*-triacetoxy-11*α*-(2-methylbutyryloxy)-urs-12-ene-3*α*,20*β*-diol demonstrated cytotoxic activity against prostate cancer cell lines (Du145—IC_50_ = 35.8 µg/mL, PC3—IC_50_ = 41.6 µg/mL), and at a concentration of 100 µg/mL reduced viability of normal prostate epithelium (PNT-2) cells by 41%.

## 1. Introduction

The over 30 plant species that are included in the genus *Maesa* are all native to tropical areas of the Old World [1]. *Maesa* was previously classified into the Myrsinaceae family, and although it has been postulated that the taxon should be the only member of the newly created Maesaceae family [2], current botanical databases [1] classify it as a member of the Primulaceae. Plants of the genus have traditionally been used as anthelminthic and antiviral remedies in both Africa and Southeast Asia [3,4,5,6,7,8,9,10]. Preparations from *Maesa lanceolata* have also been taken as nerve-stimulants and as memory restorers [5,6]. Phytochemical studies on *Maesa* spp. have led to the isolation of oleanane-type saponins, benzoquinones and flavonoids [11,12,13,14,15,16].

*Maesa membranacea* A. DC. (synonym: *Maesa subrotunda* C.Y. Wu & C. Chen) is a shrub growing wild in Cambodia, China, and Vietnam. It inhabits stream banks, hillsides, dense mixed forests, open coastal areas and damp places at elevations between 200 m and 1500 m a.s.l. [17,18]. The plant is of high cultural importance for local communities in northern and central Vietnam [19]. Traditional medicine uses the plant as a remedy against fever and hepatitis [20]. Recent research has revealed the presence of numerous phenolic constituents in leaves and stems of *M. membranacea*, including hydroxybenzoic acids (*p*-hydroxybenzoic acid, vanillic acid, protocatechuic acid) and flavonoids (kaempferol, (-)-epicatechin, kaempferol 7-*O*-*α*-rhamnopyranoside, kaempferol 3,7-di-*O*-*α*-rhamnopyranoside, kaempferol 3-*O*-*α*-arabinopyranoside-7-*O*-*α*-rhamnopyranoside). Moreover, betulinic acid was found in a methanol extract from stems of *M. membranacea* [21,22].

The objective of the present study was to investigate composition of a chloroform fraction of extract from *M. membranacea* leaves in a search for new biologically active compounds.

## 2. Results

**Figure 1:**
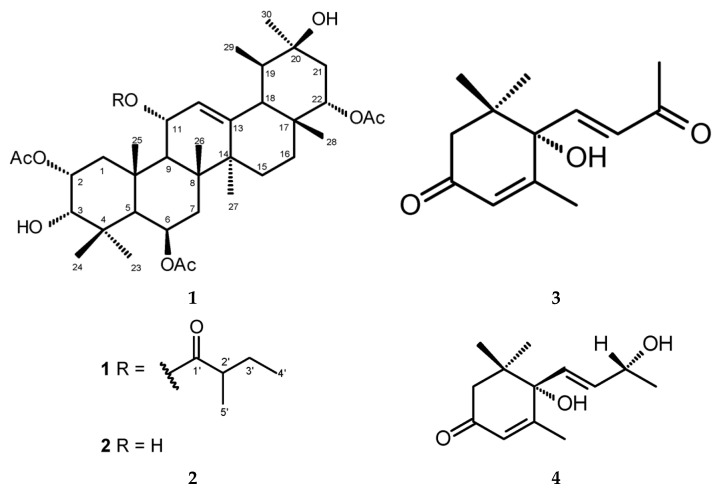
Chemical structures of 2*α*,6*β*,22*α*-triacetoxy-11*α*-(2′-methylbutyryloxy)-urs-12-ene-3*α*,20*β*-diol (**1**), 2*α*,6*β*,22*α*-triacetoxy-urs-12-ene-3*α*,11*α*,20*β*-triol (**2**), (+)-dehydrovomifoliol (**3**) and (+)-vomifoliol (**4**).

Two new ursanes (**1** and **2**) and two known apocarotenoids (**3** and **4**) (for the structures see Figure 1) were isolated from the chloroform soluble fraction of the methanolic extract from the leaves of *M. membranacea*.

### 2.1. Structure Elucidation

Compound **1** was isolated as white crystals. The HRESIMS spectrum of **1** showed an adduct ion peak *m/z* 739.4401 [M + Na]^+^ that corresponded to the molecular formula of C_41_H_64_O_10_Na (calculated mass 739.4397). The molecular formula of **1**, established as C_41_H_64_O_10_ (Figure 1), indicated ten degrees of unsaturation, attributed to five ring systems, one olefinic double bond and four ester carbonyl groups.

**Figure 2:**
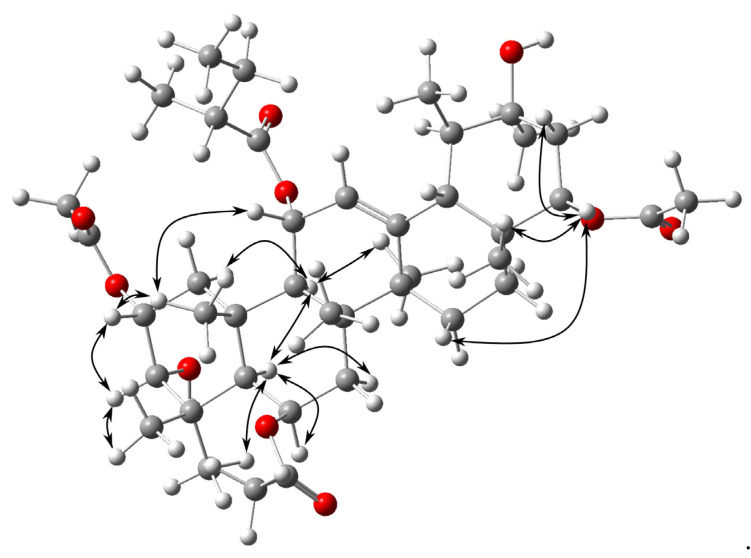
Key NOESY correlations for **1**.

The ^13^C NMR (Table 1) and HSQC spectral data indicated the presence of forty-one carbons assigned to thirteen methyl groups, six methylenes, ten methines including one olefinic and five hydroxylated or esterified groups (δ_C_ 70.05, 70.09, 70.10, 77.49 and 78.46), and twelve quaternary carbons including four carbonyls (δ_C_ 176.28, 170.27, 169.71 and 169.39) and one oxygenated quaternary carbon (δ_C_ 71.24). Signals at δ_C_ 124.82 and 142.37 indicated the presence of one double bond. The ^1^H NMR spectrum (Table 1) showed three singlets at δ_H_ 2.04, 2.08 and 2.11 (3H each) corresponding to three acetyl groups. Based on HSQC correlations, the singlets could be connected to the corresponding carbons at δ_C_ 21.20, 21.87 and 21.24, respectively. Resonances of two methyl groups (δ_H_ 0.90, t, J = 7.4 Hz, 3H, H-4′ and δ_H_ 1.10, d, J = 6.8 Hz, 3H, H-5′), one methylene group (δ_H_ 1.43, m, 1H, H-3′a, and δ_H_ 1.67, m, 1H, H-3′b) and methine group (δ_H_ 2.28, m, 1H, H-2′) corresponded to 2′-methylbutyrate substituent. According to the results of HSQC experiment, the protons showed cross peaks with carbon signals at δ_C_ 11.84, 16.15, 26.72 and 41.61, respectively. HMBC cross peaks made it possible to connect the carbonyl at δ_C_ 176.28 with 2′-methylbutyryl group and the remaining carbonyls to the respective methyls of the acetyl groups. The remaining thirty signals at ^13^C NMR spectrum, including six tertiary and two secondary methyl groups not ascribed to the four acyl functions, corresponded to a polyhydroxylated urs-12-ene triterpenoid [23,24,25]. HMBC experiment showed the correlation of methyl protons at C-23 (δ_H_ 1.10) and C-24 (δ_H_ 1.13) with C-3 (δ_C_ 77.49), which has a hydroxyl group. Correlation signals from δ_H_ 3.49 (H-3) to δ_H_ 5.29 (H-2), in ^1^H-^1^H COSY spectrum, suggested the location of acetyl group at C-2. This was supported by the HMBC correlation of quaternary carbon of acetyl group at δ_C_ 169.71 with H-2. Values of proton coupling constants of H-2 together with a small coupling constant of H-3 indicated 2α,3α orientation of the substituents at C-2 and C-3 [26]. Cross peak of C-1′at δ_C_ 176.28 and H-11 and cross peak of quaternary carbon of acetyl group at δ_C_ 169.39 and H-22, in HMBC spectrum, confirmed location of 2′-methylbutyryl substituent at C-11 and acetyl at C-22. The remaining oxygen functional groups were placed at C-6 and C-20 based on the HMBC, HSQC and ^1^H-^1^H COSY correlations. The NOESY spectrum verified the proximity H-3*β* to H-2*β*, H-16*β*, H-24*β*, OH-*β* (C-20); H-25*β* to H-2*β*, H-11*β*, H-24*β*, H-26*β* and H-22*β* to H-16*β*, H-21*β*, H-24*β*, H-28*β* as well as the proximities of H-5*α* to H-6*α*, H-7*α*, H-9*α*, H-23*α* and H-9*α* to H-1*α*, H-5*α*, H-27*α*; (Figure 2). The ^13^C NMR spectrum associated with HSQC allowed the assignments of all carbon signals of **1**, except for the quaternary carbon atoms. The HMBC spectrum confirmed the location of seven quaternary carbons at C-4, C-8, C-10, C-13, C-14, C-17 and C-20 based on the correlations from H-3*β*, H-5*α*, H-6*α*, H-7*α*, H-23*α* and H-24*β* to **C-4** as well as from H-6*α*, H-7*β*, H-9*α* and H-26*β* to **C-8**; H-1*α*, H-1*β*, H-5*α*, H-6*α*, H-7*α*, H-9*α*, H-11*β* and H-25*β* to **C-10**; H-11*β*, H-27*α* and H-30*α* to **C-13**; H-12, H-15*α*, H-18*β* and H-27*α* to **C-14**; H-18*β*, H-21*α*, H-22*β* and H-28*β* to **C-17**; H-18*β*, H-21*α*, H-22*β*, H-29*β*, H-30*α* and OH-*β* to **C-20** (Table 1).

On the basis of these results, compound **1** was deduced to be 2*α*,6*β*,22*α*-triacetoxy-11*α*-(2-methylbutyryloxy)-urs-12-ene-3*α*,20*β*-diol, a new natural product.

Compound **2** was isolated as an amorphous solid. HRESIMS spectrum of **2** showed an adduct ion peak *m/z* 655.3824 [M + Na]^+^ that corresponded to the molecular formula of C_36_H_56_O_9_Na (calculated mass 655.3822). The molecular formula of **2**, established as C_36_H_56_O_9_ (Figure 1), indicated nine degrees of unsaturation that can be attributed to five ring systems, one olefinic double bond and three ester carbonyl groups.

**Figure 3:**
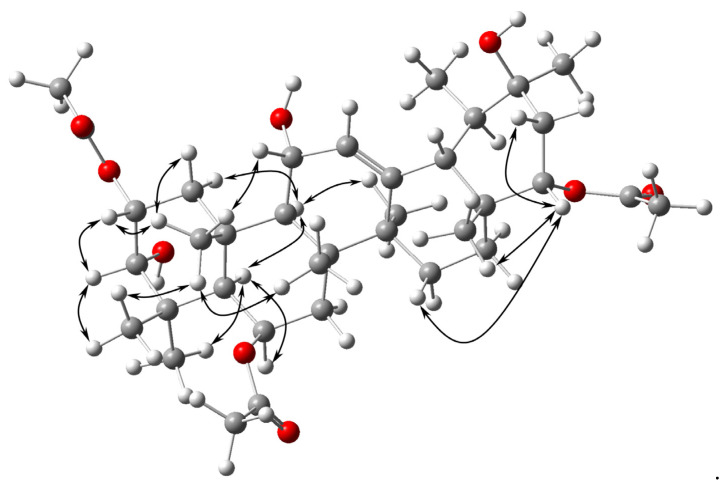
Key NOESY correlations for **2**.

The ^13^C NMR (Table 2) and HSQC spectral data indicated the presence of thirty-six carbons assigned to eleven methyl groups, five methylenes, nine methines including five hydroxylated or esterified groups (δ_C_ 67.85, 70.26, 70.37, 77.52 and 78.53) and eleven quaternary carbons including three carbonyls (δ_C_ 170.34, 170.05 and 169.40) and one oxygenated quaternary carbon (δ_C_ 71.28). Signals at δ_C_ 129.83 and 140.08 indicated the presence of the double bond. The ^1^H NMR spectrum (Table 2) showed three singlets at δ_H_ 2.07, 2.08 and 2.12 (3H each) corresponding to three acetyl groups. Based on HSQC correlations the singlets could be connected to the corresponding carbons at δ_C_ 21.28, 21.87 and 21.38, respectively. HMBC experiment showed the correlation of methyl protons at C-23 (δ_H_ 1.11) and C-24 (δ_H_ 1.15) with C-3 (δ_C_ 77.52) which has a hydroxyl substituent. Correlation signals from δ_H_ 3.49 (H-3) to δ_H_ 5.33 (H-2), in ^1^H-^1^H COSY spectrum, suggested the location of acetyl group at C-2. The location was confirmed by the HMBC spectrum. Cross peak of the quaternary carbon of acetyl group at δ_C_ 169.40 and H-22, in HMBC spectrum, also confirmed the placement of acetyl at C-22. The remaining oxygen functional groups were situated at C-6, C-11 and C-20 based on the HMBC, HSQC and ^1^H-^1^H COSY correlations. The NOESY spectrum verified the proximity H-3*β* to H-2*β*, H-24*β*; H-25*β* to H-1*β*, H-2*β*, H-11*β*, H-24*β*, H-26*β* and H-22*β* to H-16*β*, H-21*β*, H-24*β*, H-28*β* as well as the proximities of H-5*α* to H-6*α*, H-9*α*, H-23*α* and H-9*α* to H-1*α*, H-5*α*, H-27*α*; (Figure 3). The ^13^C NMR spectrum associated with HSQC allowed the assignments of all carbon signals of **2** except for the quaternary carbon atoms. The HMBC spectrum confirmed the location of seven quaternary carbons at C-4, C-8, C-10, C-13, C-14, C-17 and C-20 based on the correlations from H-3*β*, H-5*α*, H-7*α*, H-7*β*, H-23*α* and H-24*β* to **C-4** as well as from H-9*α*, H-11*β*, H-15*β*, H-26*β* and H-27*α* to **C-8**; H-1*α*, H-1*β*, H-2*β*, H-7*α*, H-7*β*, H-9*α* and H-25*β* to **C-10**; H-18*β* and H-27*α* to **C-13**; H-12, H-15*β* and H-18*β* to **C-14**; H-18*β*, H-21*α*, H-21*β*, H-22*β* and H-28*β* to **C-17**; H-22*β*, H-29*β* and H-30*α* to **C-20** (Table 2).

On the basis of these results, compound **2** was deduced to be 2*α*,6*β*,22*α*-triacetoxy-urs-12-ene-3*α*,11*α*,20*β*-triol, a new natural product.

The known compounds **3** and **4** were identified as (+)-dehydrovomifoliol and (+)-vomifoliol, respectively, by comparison of their spectral data with that found in the literature [27,28].

### 2.2. Cytotoxic Activity

Cytotoxicities of **1** and **2**, at a dose range of 5–100 μg/mL, were tested against three panels of human cancer and normal cells (see Table 3 and Table S1 in the Supplementary Materials). In the skin panel, both compounds exerted weak cytotoxicity towards HTB140 and A375 melanoma cells (at 100 μg/mL of **1**, viability of the most susceptible cell line HTB140 exceeded 45%) and keratinocytes (100 μg/mL, over 54% viable cells), after 24 h treatment. Compound **2**, in general, was less active than **1**, against all cancer cell lines used in the study. The two colon cancer cell lines used in the experiment demonstrated different sensitivities to the treatment with **1**. The triterpene was modestly toxic to Caco-2 cells (IC_50_—35.7 µg/mL) whereas the line HT29 was less susceptible (100 μg/mL, over 55% viable cells). Prostate cancer cell lines of different metastatic potential were used for the cytotoxicity assessment. Compound **1** was less effective (IC_50_—41.6 µg/mL) against PC3 cells (with high metastatic potential) than against DU145 cells (IC_50_—35.8 µg/mL). The normal prostate epithelial cells PNT-2 showed over 59% viability after 24 h of treatment with 100 µg/mL of **1** and over 93% viability after the treatment with 100 μg/mL of **2**, which indicates selective activity of the compounds. Though IC_50_ values for the compound **2** exceeded the dose of 50–100 μg/mL, the compound demonstrated cytotoxicity profile similar to that of **1** (Table S1) with the highest activity towards Caco-2 and PC3 cells. Similar to **1**, compound **2** was less active against prostate normal epithelial cells than against the DU 145 and PC3 prostate cancer cells (over 57% and 47% viable cells, respectively, after the treatment with 100 μg/mL of **2**).

## 3. Discussion

Triterpenoids of ursane and oleanane type are ubiquitous plant constituents. According to the published phytochemical studies, plants from the Primulaceae family synthesize oleanane derivatives almost exclusively. In fact, we found only two papers that dealt with ursane-type triterpenoid isolation from the plants included in the family. The first one was on saponin—clethroidoside H separation from *Lysimachia clethroides* Duby [29], and the second was on extraction of ursolic acid from the stem wood of *Maesa lanceolata* Forssk. [30]. Ursanes are not rare in plants from the other families of Ericales (e.g., Actinidiaceae, Ericaceae and Lecythidaceae) and the hydroxyl groups at 2*α*, 3*α* and 6*β* are not unique there [31,32,33]. Polyhydroxylated ursanes with substitution pattern similar to **1** were described earlier as constituents of *Salvia argentea* L. (Lamiaceae) [22] and *Siphonodon celastrineus* Griff. (Celastraceae) [34]. In both cases, however, the hydroxyl group at C-3 was *β* oriented. Our results clearly indicate α-orientation of the hydroxyl group in **1** and **2**.

Oleanane-type triterpene saponins have hitherto been the only triterpene saponins isolated from the plants of *Maesa* spp. [11,12,13,30,35,36,37,38,39,40]. All of the compounds share the oleanane skeleton hydroxylated at 3*β*, 16*α*, 21*β* and/or 22*α* and 28*α*. To our knowledge, ursolic acid is the only triterpene with the ursane-type skeleton that has been found in *Maesa* spp. before. Thus, the presence of **1** and **2** seems to be a good taxonomic marker of *M. membranacea*.

Cytotoxicity of **1** and **2** was tested against three panels of human cancer and normal cells (Table 3 and Table S1). In general, compound **1** demonstrated higher activity against all cell lines used in the study. As the only difference in structure between **1** and **2** was the presence or absence of 2-methylbutyryl group bonded to the oxygen at C-11, it may suggest that the lower polarity of the molecule and/or its shape is responsible for the more pronounced cytotoxicity of **1**. Selectivity in the cytotoxic effect of **1** towards the prostate cancer cells as well as the activity of the triterpene against doxorubicin-resistant cells (PC3 line) is worth noting.

Ursane-type triterpenes with multiple oxygen functionalities frequently demonstrated cytotoxic activity towards human and murine cancer cells in vitro [41,42,43,44,45]. The described IC_50_ values vary broadly (5.7–57.0 µM), depending on the structure of the investigated compound, the cell line used, and the time of exposure applied (24–96 h). This makes the direct comparison of the results difficult. Some remarks on relationships between the chemical structure and the biological activity of substituted ursolic acid derivatives have been summarized by Sommerwerk et al. [45]. They concluded that ursanes were, in general, less active than oleananes with the corresponding substitution pattern. The two acetyl groups at C-2 and C-3 were in favor of cytotoxicity; however, their optimum configuration should be 2*β*,3*β* (contrary to 2*α*,3*α* in **1** and **2**).

Apocarotenoids: (+)-dehydrovomifoliol (**3**) and (+)-vomifoliol (**4**) are biologically active compounds frequently found in the aerial parts of plants from different taxonomic groups. However, their presence in *Maesa* ssp. has not been described yet.

## 4. Materials and Methods

### 4.1. General Experimental Procedures

NMR spectra were recorded in CDCl_3_ on a Bruker AVANCE III HD 400 (resonance frequency 400.17 MHz for ^1^H and 100.63 MHz for ^13^C) spectrometer (Bruker Corp., Billerica, MA, USA). High resolution mass spectra were obtained in the positive ion mode using MaldiSYNAPT G2-S HDMS (Waters Inc., Milford, MA, USA) mass spectrometer equipped with an electrospray ion source and Q-TOF type mass analyzer. Optical rotation was determined in CDCl_3_ on a PolAAr31 polarimeter (Optical Activity Ltd., Huntingdon, England). RP-HPLC separations were performed using an Agilent 1200 Series HPLC system (Agilent Technologies Inc., Santa Clara, CA, USA) equipped with a diode array detector. Analytical chromatographic separations were carried out on a Kinetex XB-C18 column (4.6 × 250 mm, 5 μm total particle size; Phenomenex Inc., Torrance, CA, USA). Semipreparative RP-HPLC was conducted on a Vertex Plus Eurospher II 100-5 C18 column (250 × 8 mm; Knauer GmbH, Berlin, Germany), with an isocratic elution, using methanol–water (MeOH-H_2_O) mixtures of different polarity. Conventional column chromatography (CC) was carried out on Silica gel 60 (0.063–0.2 mm, Merck KGaA, Darmstadt, Germany). Thin-layer chromatography (TLC) separations were performed using precoated plates (Silica gel 60 without fluorescence indicator, Art. No 5553, Merck, Darmstadt, Germany). Solvents of analytical grade were purchased from Avantor Performance Materials S.A. (Gliwice, Poland). Water was purified by a Mili-Q system (Milipore Corp., Bedford, MA, USA). MeOH and MeCN of HPLC grade were bought from Merck (Darmstadt, Germany).

### 4.2. Plant Material

Leaves of *M. membranacea* A. DC. were collected from the Kontum province (Vietnam), and were taxonomically verified by Dr. Nguyen Quoc Binh from the Vietnam Museum of Nature of the Vietnam Academy of Science and Technology (VAST). A voucher specimen (VN-2292) has been deposited in the Institute of Marine Biochemistry VAST in Hanoi.

### 4.3. Extraction and Isolation of **1**–**4**

Coarsely ground dried leaves of *M. membranacea* (665 g) were extracted, as previously described [22], with 80% MeOH (5 × 4 L) in room temperature. The obtained extracts were concentrated in vacuo to yield 193.5 g of an oily residue. The residue was suspended in water (1 L) and subsequently partitioned with solvents of increasing polarity. The chloroform fraction of the extract (5.9 g) was subjected to CC over silica gel (104 g) using mobile phase gradients of ethyl acetate (EtOAc) in hexane (up to 100% EtOAc; fractions 1–396) and MeOH in EtOAc (up to 20% MeOH; fractions 397–424). The separated fractions (50 mL each) were monitored by TLC (supported by RP-HPLC, if necessary) and the relevant ones were combined. Fractions 117–122 (eluted with hexane-EtOAc; 3:2, *v*/*v*), after purification by preparative TLC (precoated TLC plates, solvent system: hexane-EtOAc, 3:2, *v*/*v*), yielded **3** (3.6 mg). Further elution of the column with hexane-EtOAc (3:2, *v*/*v*) gave fractions 130–138 and 186–198. The fractions 130–138 were subsequently subjected to semipreparative HPLC on a Vertex Plus Eurospher II 100-5 C18 column (mobile phase: MeOH-H_2_O; 9:1, *v*/*v*, 2 mL/min) to yield **1** (t_R_ = 9.7 min, 78.6 mg). The fractions 186–198 were further separated by semipreparative HPLC using MeOH-H_2_O (3:2, *v*/*v*; flow rate: 2 mL/min) to give **4** (t_R_ = 31 min, 5.7 mg). Fractions 296–299 (eluted with hexane-EtOAc; 1:1, *v*/*v*) were further separated by preparative TLC (CHCl_3_–MeOH; 19:1, *v*/*v*) to give **2** (8.5 mg).

#### Characterization of the Isolated Compounds **1**–**4**

2*α*,6*β*,22*α*-Triacetoxy-11*α*-(2-methylbutyryloxy)-urs-12-ene-3*α*,20*β*-diol (**1**). White crystals: [α]_D_^28^: −58.8° (c = 0.33, CHCl_3_); UV (MeCN-H_2_O) λ_max_ 203 nm; ^1^H- and ^13^C-NMR: Table 1, Supplementary Material Figures S2–S7; HRESIMS (pos. mode) *m/z*: 739.4401 [C_41_H_64_O_10_Na]^+^; calc. 739.4397, Supplementary Material Figure S1.

2*α*,6*β*,22*α*-Triacetoxy-urs-12-ene-3*α*,11*α*,20*β*-triol (**2**). White, amorphous solid: [α]_D_^28^: 0° (c = 2.67, CHCl_3_); UV (MeCN-H_2_O) *λ*_max_ 205 nm; ^1^H- and ^13^C-NMR: Table 2, Supplementary Material Figures S9–S14; HRESIMS (pos. mode) *m/z*: 655.3824 [C_36_H_56_O_9_Na]^+^; calc. 655.3822, Supplementary Material Figure S8.

(+)-Dehydrovomifoliol = (6*S*)-6-hydroxy-3-oxo-*α*-ionone (**3**). Amorphous solid: [α]_D_^28^: + 37.9° (c = 1.17, CHCl_3_); ^1^H-NMR, Supplementary Material Figure S15.

(+)-Vomifoliol = (6*S*,9*R*) -6-hydroxy-3-oxo-*α*-ionol (**4**). Amorphous solid: [α]_D_^28^: + 95.2° (c = 1.60, CHCl_3_); ^1^H-NMR, Supplementary Material Figure S16.

### 4.4. Cell Culture and Cytotoxicity Assessment

Cytotoxic activity was tested on human cancer and normal cells, grouped in three panels, namely: prostate, skin and gastrointestinal. The prostate panel consisted of prostate cancer cell lines Du145 (ATCC HTB-81) and PC3 (ATCC CRL-1435), and prostate epithelial cells PNT-2 (ECACC 95012613). Melanoma cell lines A375 (ATCC CRL-1619) and HTB140 (ATCC Hs 294T) together with skin keratinocytes HaCaT (obtained as a kind gift of prof. Marta Michalik, Department of Cell Biology, Jagiellonian University, Kraków, Poland) were included into the skin panel. The gastrointestinal panel of cells comprised colon cancer cell lines Caco-2 (ATCC HTB-37) and HT29 (ATCC HTB-38). Du145 cells were grown in Modified Eagle’s Medium with low (1.0 g/L) glucose concentration, HT29, PC3 and PNT-2 cells in Dulbecco’s Modified Eagle’s Media: F12 HAM Nutrient Mixture, Caco-2 in Modified Eagle’s Medium with NEAA (no-essential amino acids) while melanoma cells and keratinocytes were maintained in Modified Eagle’s Medium with high (4.5 g/L) glucose concentration. The culture media (all supplied by Sigma-Aldrich Co.; St. Louis, MO, USA) contained antibiotics and 10% fetal bovine serum (FBS). All cultures were maintained at 37 °C, in a humidified, 5% CO_2_ containing, atmosphere.

The examined triterpenes were diluted in the culture media from freshly made stock solutions in MeOH (10 mg/mL) to the working concentrations (from 0 to 100 μg/mL).

Cell viability was determined as it was described previously [46]. Cells suspended in the nutrient medium were transferred into 96-well microtiter plates (density 1.5 × 10^4^ per well), and preincubated for 24 h (37 °C, 5% CO_2_). Then, the culture medium was replaced with the medium containing different concentrations of **1** or **2** (1–100 μg/mL). After 24 h of incubation, viability of the cells was determined using colorimetric lactate dehydrogenase (LDH) assay, in comparison to the controls to which corresponding aliquots of MeOH diluted with culture media were added. Cells grown in the medium without the tested compounds were used as control I (negative) and the positive control (control II) was obtained by incubation of the cells in the medium containing 1% Triton X-100. LDH released from the damaged cells into the cell culture medium was quantified by measuring the absorbance at 490 nm using Synergy II Biotek (BioTek Instruments, Winooski, VT, USA) microplate reader. Cytotoxicities of the examined compounds were calculated as follows: [(absorbance of the tested sample − absorbance of control I)/(absorbance of control II − absorbance of control I)] × 100. Results were means of three independent measurements (± SD). Doxorubicin (Ebewe Pharma GmbH., Unterach, Austria) was used as a reference cytostatic drug. The IC_50_ values were determined by plotting the percentage viability of the cells versus concentration and the adequate calculations made using AAT Bioquest website program (https://www.aatbio.com/tools/ic50-calculator, accessed on 29 October 2021).

## 5. Conclusions

The isolated polyesterified triterpenes represent the structural type of the compounds with unique substitution pattern. Polyfunctionalized triterpenes of ursane-type have not been previously found in *Maesa* spp. and are very rare in the Primulaceae. They seem to be good systematic markers of *M. membranacea*.

2*α*,6*β*,22*α*-Triacetoxy-11*α*-(2-methylbutyryloxy)-urs-12-ene-3*α*,20*β*-diol demonstrated moderate but selective cytotoxicity towards prostate cancer cell lines (including doxorubicin resistant PC3 cells) and moderate activity towards the Caco 2 colon cancer cells. Normal prostate epithelial cells PNT-2 were less sensitive to the investigated triterpenoid.

## Figures and Tables

**Table 1:** ^1^H NMR (400.17 MHz) and ^13^C NMR (100.63 MHz) data of compound **1** in CDCl_3_.

Position	δ_H_ (ppm), *J* (Hz)	δ_C_ (ppm)	HMBC (H→C)
1α	1.74 m	41.38	C-2, C-3, C-9, C-10, C-25
1β	1.50 m		C-2, C-3, C-5, C-10, C-25
2β	5.29 ddd (9.6, 4.8, 4.4)	(70.05/70.09/70.10) ^d^	C-OAc
3β	3.49 brs	77.49	C-1, C-2, C-4, C-5, C-23, C-24
4	-	38.69	-
5α	1.63 brs	46.95	C-4, C-10, C-24, C-25
6α	5.50 brs	(70.05/70.09/70.10) ^d^	C-4/10, C-8
7α	1.70 m	36.44	C-4/10, C-6, C-9
7β	1.79 m		C-8
8	-	42.50	-
9α	2.17 d (8.8)	51.50	C-8, C-10, C-11, C-25, C-26
10	-	38.76	-
11β	5.65 dd (8.8, 3.2)	(70.05/70.09/70.10) ^d^	C-9, C-10, C-12, C-13, C-27, C-1′
12	5.33 d (3.2)	124.82	C-9, C-11, C-14, C-18, C-19, C-27
13	-	142.37	-
14	-	42.60	-
15α	1.83 m	26.35	C-14, C-18
15β	1.08 m		-
16α	1.13 ^a^ m	26.72 ^e^	-
16β	1.88 m		C-28
17	-	36.91	-
18β	1.96 m	48.89	C-14, C-15, C-17, C-20, C-28, C-29
19α	1.77 m	40.60	C-21, C-29
20	-	71.24	-
21α	1.84 m	38.56	C-17, C-20, C-22
21β	1.92 m		C-20
22β	4.88 brs	78.46	C-16, C-17, C-18, C-20, C-21, C-OAc
23α	1.10 ^b^ s	28.44	C-3, C-4, C-5, C-24
24β	1.13 ^a^ s	23.14	C-3, C-4, C-5, C-23
25β	1.55 s	19.45	C-1, C-5, C-9, C-10, C-26
26β	1.29 s	18.61	C-7, C-8, C-9
27α	1.23 s	23.07	C-13, C-14, C-15
28β	0.81 s	21.11	C-16, C-17, C-18, C-22
29β	0.91 ^c^ d (6.4)	12.40	C-18, C-19, C-20
30α	1.19 s	29.07	C-13, C-19, C-20, C-21, C-22
OH (C-20)	2.77 s	-	C-20, C-21, C-30
OAc (C-22)_CO	-	169.39	-
OAc (C-2/6)_CO	-	169.71	-
OAc (C-2/6)_CO	-	170.27	-
OAc_CH_3_	2.04 s	21.20	OAc (C-2/6)_CO, C-2/6
OAc_CH_3_	2.08 s	21.87	OAc (C-2/6)_CO, C-2/6
OAc_CH_3_	2.11 s	21.24	OAc (C-22)_CO, C-22
1′	-	176.28	-
2′	2.28 m	41.61	C-1′, C-3′, C-4′, C-5′
3′a	1.43 m	26.72 ^e^	C-1′, C-2′, C-4′, C-5′
3′b	1.67 m		C-1′, C-2′, C-4′, C-5′
4′	0.90 ^c^ t (7.4)	11.84	C-2′, C-3′
5′	1.10 ^b^ d (6.8)	16.15	C-1′, C-2′, C-3′

^a, b, c^ signals overlapped, ^d, e^ signals interchangeable.

**Table 2:** ^1^H NMR (400.17 MHz) and ^13^C NMR (100.63 MHz) data of compound **2** in CDCl_3_.

Position	δ_H_ (ppm), *J* (Hz)	δ_C_ (ppm)	HMBC (H→C)
1α	1.74 m	42.11	C-2, C-9, C-10, C-25
1β	2.33 dd (12.8, 4.0)		C-2, C-3, C-5, C-10, C-25
2β	5.33 ^a^ m	70.37	C-9, C-10, C-OAc
3β	3.49 brs	77.52	C-1, C-2, C-4, C-5, C-23, C-24
4	-	38.73	-
5α	1.62 brs	47.17	C-4, C-24, C-25
6α	5.49 brs	70.26	-
7α	1.69 m	36.72	C-4/10, C-26
7β	1.77 m		C-4/10
8	-	42.51	-
9α	1.81 ^b^ m	54.64	C-8, C-10, C-11, C-25/26
10	-	38.87	-
11β	4.46 dd (8.8; 2.6)	67.85	C-8, C-9
12	5.34 ^a^ d (3.1)	129.83	C-9, C-11, C-14, C-18, C-27
13	-	140.08	-
14	-	42.89	-
15α	1.83 m	26.20	-
15β	1.07 m		C-8, C-14-
16α	1.13 ^c^ m	26.82	-
16β	1.87 m		-
17	-	37.09	-
18β	1.94 ^d^ m	49.10	C-12, C-13, C-14, C-15, C-17, C-28
19α	1.80 ^b^ m	40.33	C-18, C-29
20	-	71.28	-
21α	1.88 m	38.60	C-17
21β	1.93 ^d^ m		C-17, C-19
22β	4.90 brs	78.53	C-16, C-17, C-18, C-20, C-21, C-OAc
23α	1.11 s	28.69	C-3, C-4, C-5, C-24
24β	1.15 ^c^ s	23.33	C-3, C-4, C-5, C-23
25β	1.59 s	18.96	C-1, C-5, C-9, C-10
26β	1.26 s	18.87	C-7, C-8, C-9
27α	1.21 ^e^ s	23.57	C-8, C-13, C-15
28β	0.81 s	21.20	C-16, C-17, C-18, C-22
29β	0.91 d (6.4)	12.53	C-18, C-19, C-20, C-21, C-30
30α	1.21 ^e^ s	29.07	C-19, C-20, C-21, C-22
OH (C-20)	2.80 brs	-	-
OAc (C-22)_CO	-	169.40	-
OAc (C-2/6)_CO	-	170.05	-
OAc (C-2/6)_CO	-	170.34	-
OAc_CH_3_	2.07 s	21.87	OAc (C-2/6)_CO, C-2/6
OAc_CH_3_	2.08 s	21.38	OAc (C-2/6)_CO, C-2/6
OAc_CH_3_	2.12 s	21.28	OAc (C-22)_CO, C-22

^a, b, c, d^ signals overlapped, ^e^ signals interchangeable.

**Table 3:** Cytotoxicities of **1** and **2** against human normal and cancer cell lines, after the 24 h treatment (5–100 μg/mL).

Compound	IC_50_ (μg/mL)							
	Prostate Normal and Cancer Cells ^a^			Keratinocytes and Melanoma Cells ^b^			Colon Cancer ^c^	
	PNT-2	DU145	PC3	HaCaT	A375	HTB140	HT29	Caco-2
**1**	>100	35.83 (50.00) ^d^	41.64 (58.15) ^d^	>100	>100	>50	>100	35.65 (49.79) ^d^
**2**	>100	>100	>50	>100	>100	>100	>100	>50
**Doxorubicin**	1.38	3.18	>50	4.68	0.59	5.71	1.53	3.44

^a^ PNT-2—human normal prostate epithelium cells, DU145—human prostate carcinoma cells, PC3—human prostate adenocarcinoma cells. ^b^ HaCaT—human immortalized keratinocytes, A375—human malignant melanoma cells, HTB140—human melanoma cells. ^c^ HT29—human colon adenocarcinoma cells, Caco-2—human colon adenocarcinoma cells. ^d^ IC_50_ (µM).

## Data Availability

The raw data that support the findings of this study are available from the authors (A.G., K.M., J.M., A.S., T.N.L.), upon reasonable request.

## Supplementary Materials

The following are available online, Figures S1–S14: HR ESIMS and NMR spectra of **1** and **2**, Figures S15 and S16: ^1^H-NMR spectra of **3** and **4**, Table S1: Viability of human normal and cancer cell lines treated for 24 h with 5–100 μg/mL of **1** or **2**.

## Sample Availability

Samples of the compounds are not available from the authors.

## Abbreviations

1D NMR	One-dimensional nuclear magnetic resonance spectroscopy
2D NMR	Two-dimensional nuclear magnetic resonance spectroscopy
CC	Conventional column chromatography
CDCl_3_	Deuterated chloroform
COSY	Correlation spectroscopy (2D NMR experiment)
EtOAc	Ethyl acetate
HMBC	Heteronuclear multiple bond correlation (2D NMR experiment)
HRESIMS	High-resolution electrospray ionization mass spectrometry
HSQC	Heteronuclear single quantum coherence (2D NMR experiment)
LDH	Lactate dehydrogenase
MeCN	Acetonitrile
MeOH	Methanol
NOESY	Nuclear Overhauser effect spectroscopy (2D NMR experiment)
Q-TOF	Quadrupole time-of-flight
RP-HPLC	Reversed-phase high-performance liquid chromatography
TLC	Thin-layer chromatography
