# Supplementary material for: New Polyesterified Ursane Derivatives from Leaves of Maesa membranacea and Their Cytotoxic Activity
Source: Molecules. 2021 Nov 20;26(22):7013. doi: 10.3390/molecules26227013 (PMC8619920; doi:10.3390/molecules26227013)
Supplement: Supplementary file 1 [file molecules-26-07013-s001.zip › molecules-1463704-supplementary.pdf]

SUPPLEMENTARY MATERIAL

New polyesterified ursane derivatives from leaves of *Maesa membranacea* and their cytotoxic activity

Klaudia Michalska [a,1], Agnieszka Galanty [2], Thanh Nguyen Le [a,3], Janusz Malarz [1], Nguyen Quoc Vuong [3], Cuong Van Pham [3] and Anna Stojakowska [a,1*]

List of supporting information

Figure S1. HR ESIMS spectrum of compound **1**.

Figure S2. ^{1}H NMR spectrum of compound **1**.

Figure S3. ^{13}C NMR spectrum of compound **1**.

Figure S4. ^{1}H-^{1}H COSY spectrum of compound **1**.

Figure S5. NOESY spectrum of compound **1**.

Figure S6. HSQC spectrum of compound **1**.

Figure S6a. Expansion of HSQC spectrum of compound **1**.

Figure S7. HMBC spectrum of compound **1**.

Figure S7a. Expansion of HMBC spectrum of compound **1**.

Figure S8. HR ESIMS spectrum of compound **2**.

Figure S9. ^{1}H NMR spectrum of compound **2**.

Figure S10. ^{13}C NMR spectrum of compound **2**.

Figure S11. ^{1}H-^{1}H COSY spectrum of compound **2**.

Figure S12. NOESY spectrum of compound **2**.

Figure S13. HSQC spectrum of compound **2**.

Figure S13a. Expansion of HSQC spectrum of compound **2**.

Figure S14. HMBC spectrum of compound **2**.

Figure S14a. Expansion of HMBC spectrum of compound **2**.

Figure S15. ^{1}H NMR spectrum of compound **3**.

Figure S16. ^{1}H NMR spectrum of compound **4**.

Table S1. Viability of human normal and cancer cell lines treated for 24 h with 5-100 μg/mL of **1** or **2** (values are means of three measurements ± SD).

Single Mass Analysis

Tolerance = 5.0 mDa / DBE: min = -0.5, max = 100.0

Element prediction: Off

Number of isotope peaks used for i-FIT = 2

Monoisotopic Mass, Even Electron Ions

126 formula(e) evaluated with 1 results within limits (all results (up to 1000) for each mass)

Elements Used:

C: 0-100 H: 0-200 O: 0-15 Na: 1-1

Mass	Calc. Mass	mDa	PPM	DBE	Formula	i-FIT	i-FIT Norm	Fit Conf %	C	H	O	Na
739.4401	739.4397	0.4	0.5	9.5	C41 H64 O10 Na	184.1	n/a	n/a	41	64	10	1

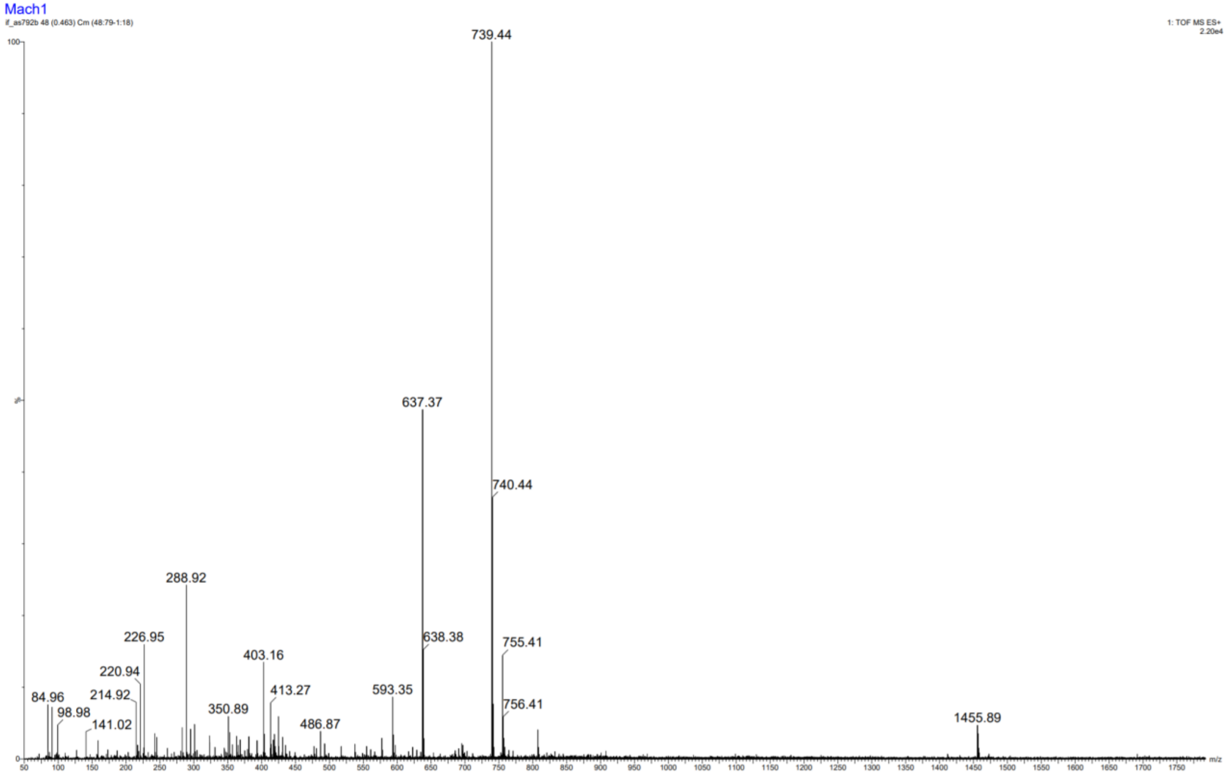

Figure S1. HR ESIMS spectrum of compound **1**.

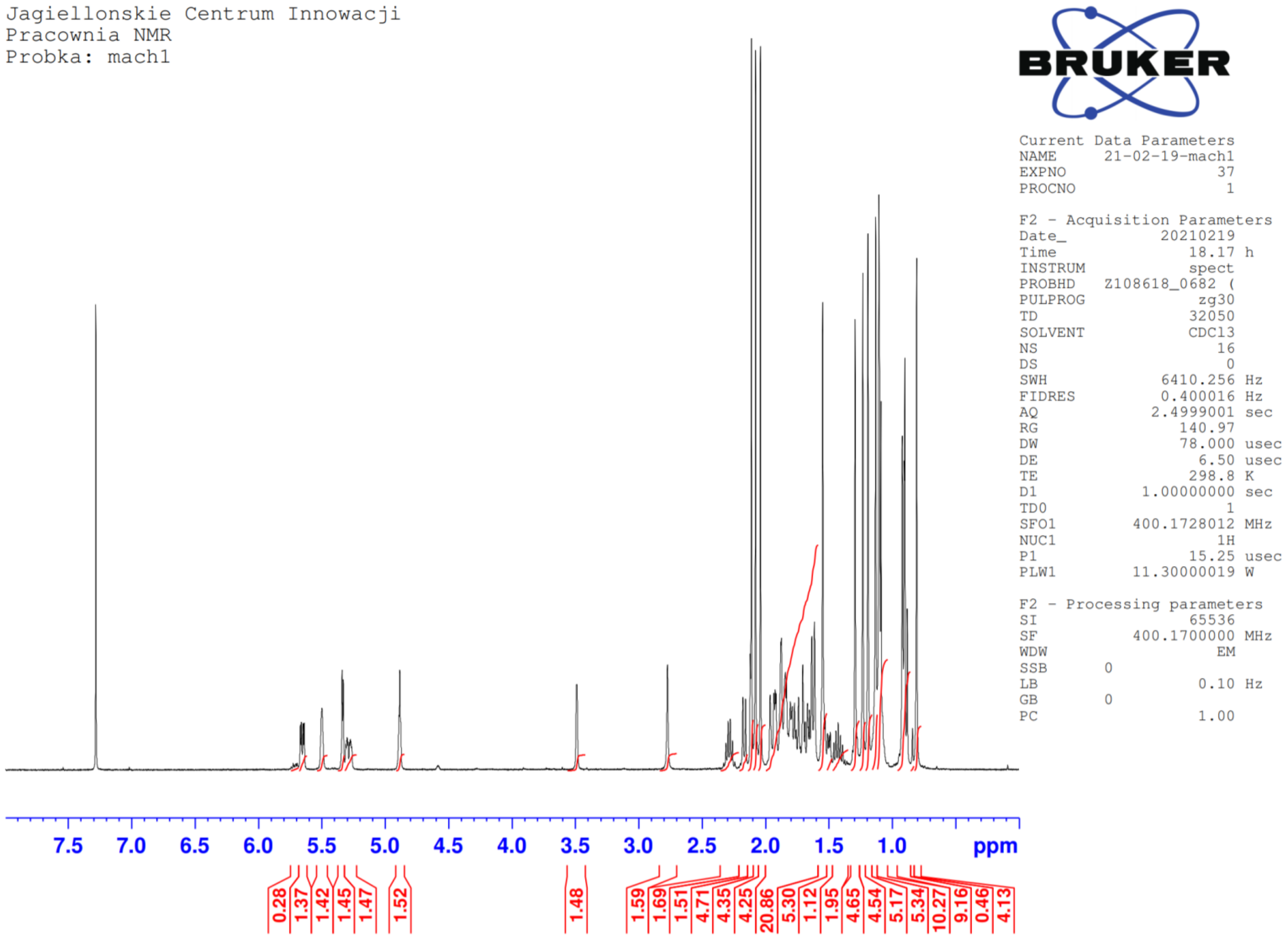

Figure S2. ^{1}H NMR spectrum of compound **1**.

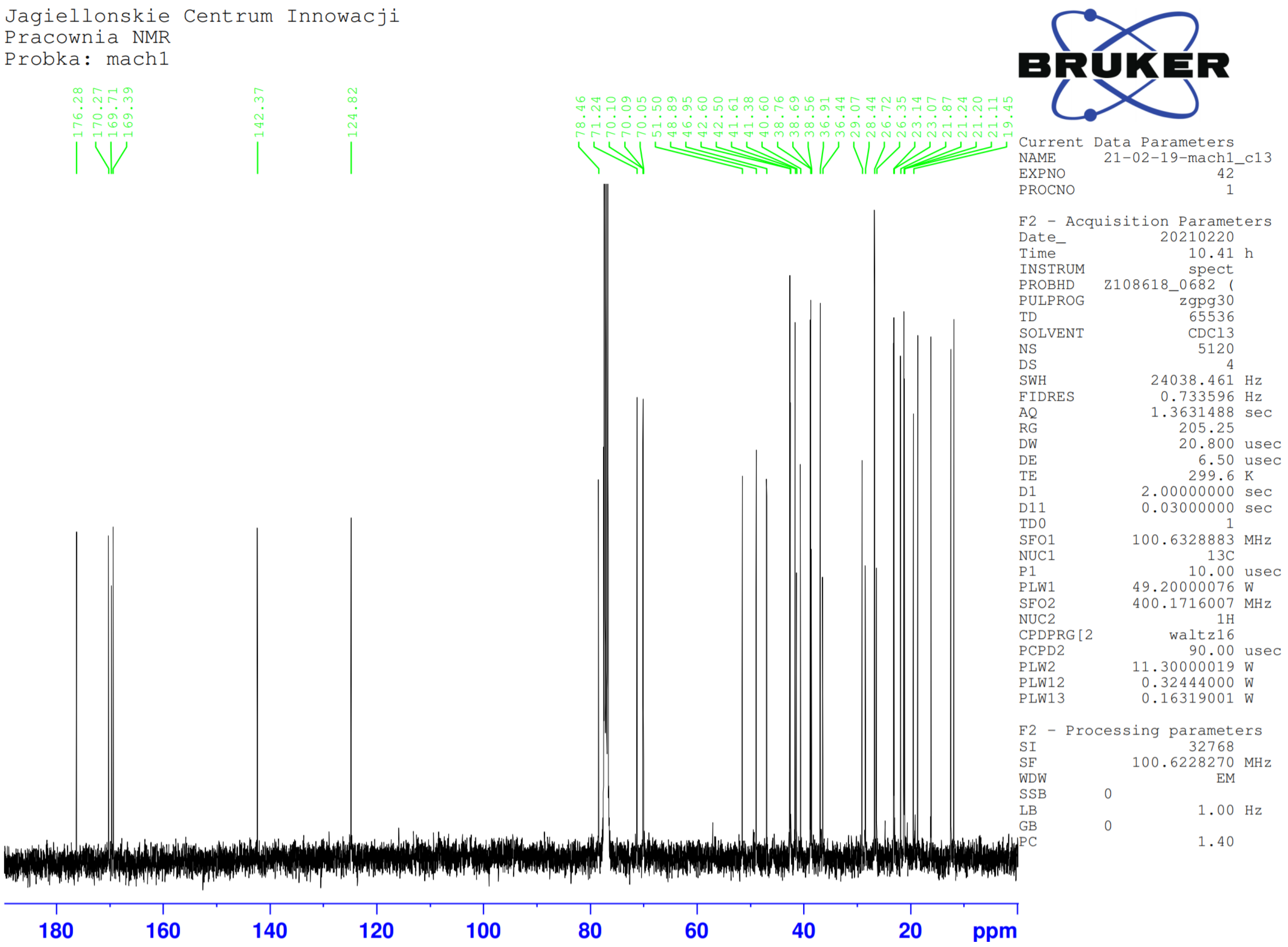

Figure S3. ^{13}C NMR spectrum of compound **1**.

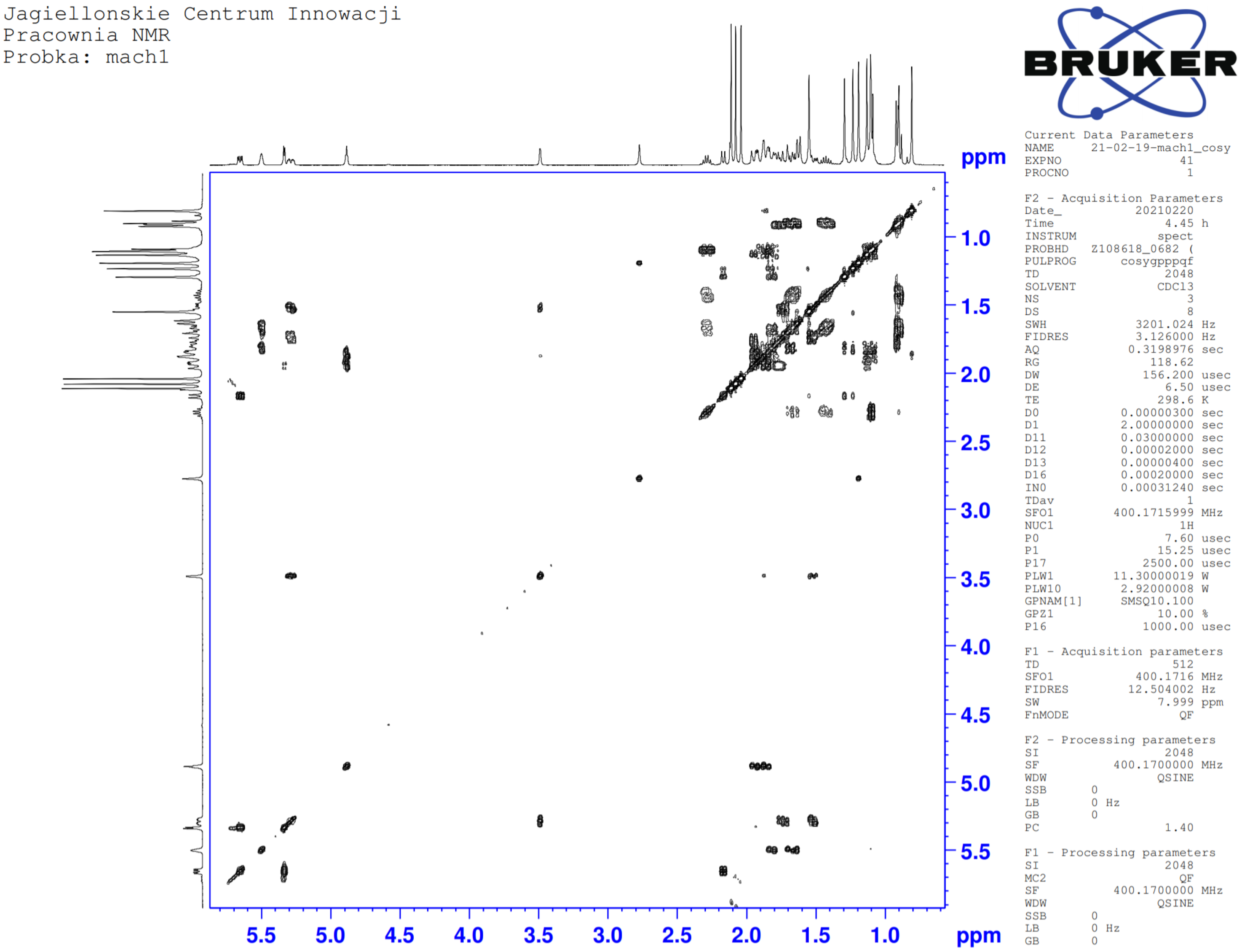

Figure S4. ^{1}H-^{1}H COSY spectrum of compound **1**.

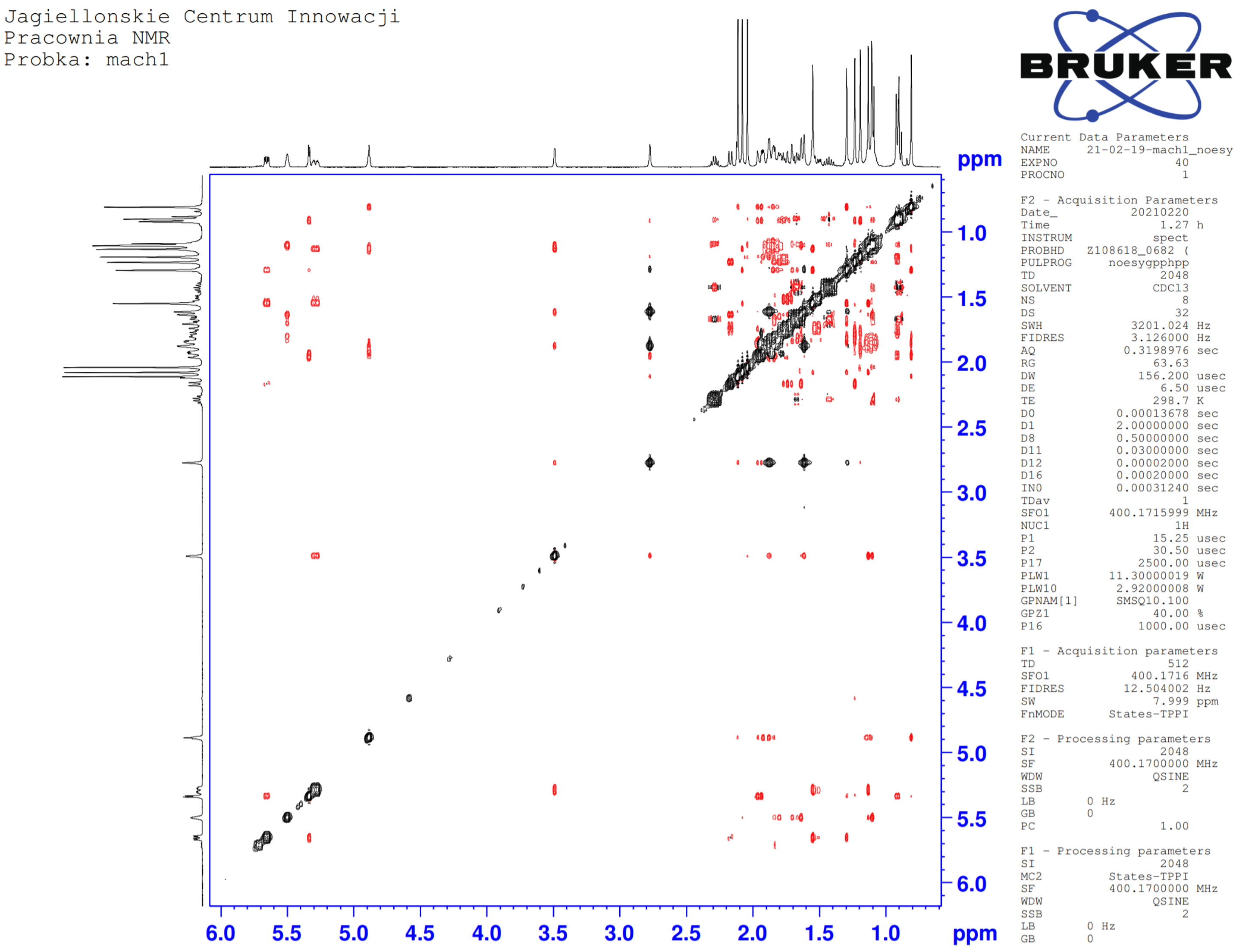

Figure S5. NOESY spectrum of compound **1**.

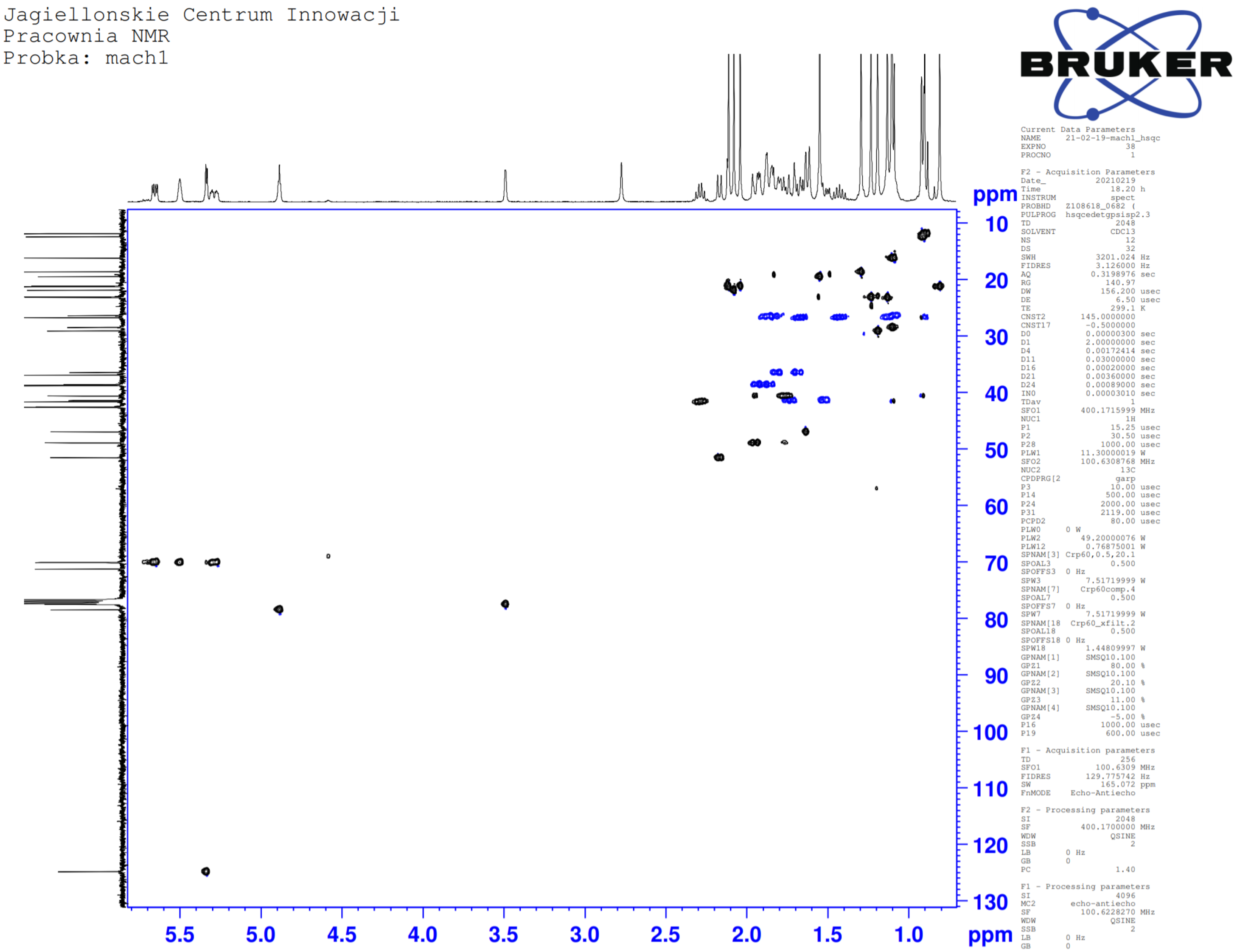

Figure S6. HSQC spectrum of compound **1**.

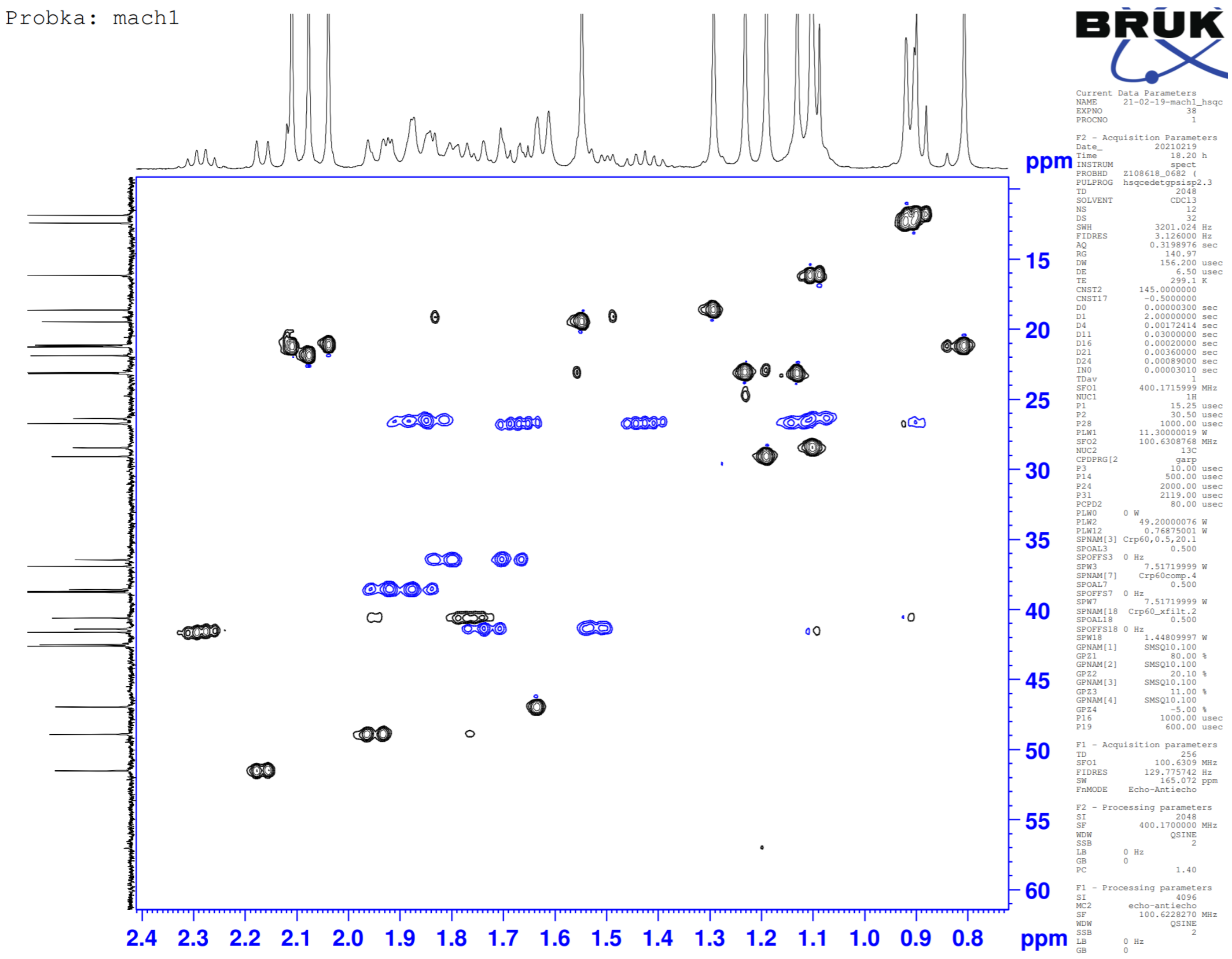

Figure S6a. Expansion of HSQC spectrum of compound **1**.

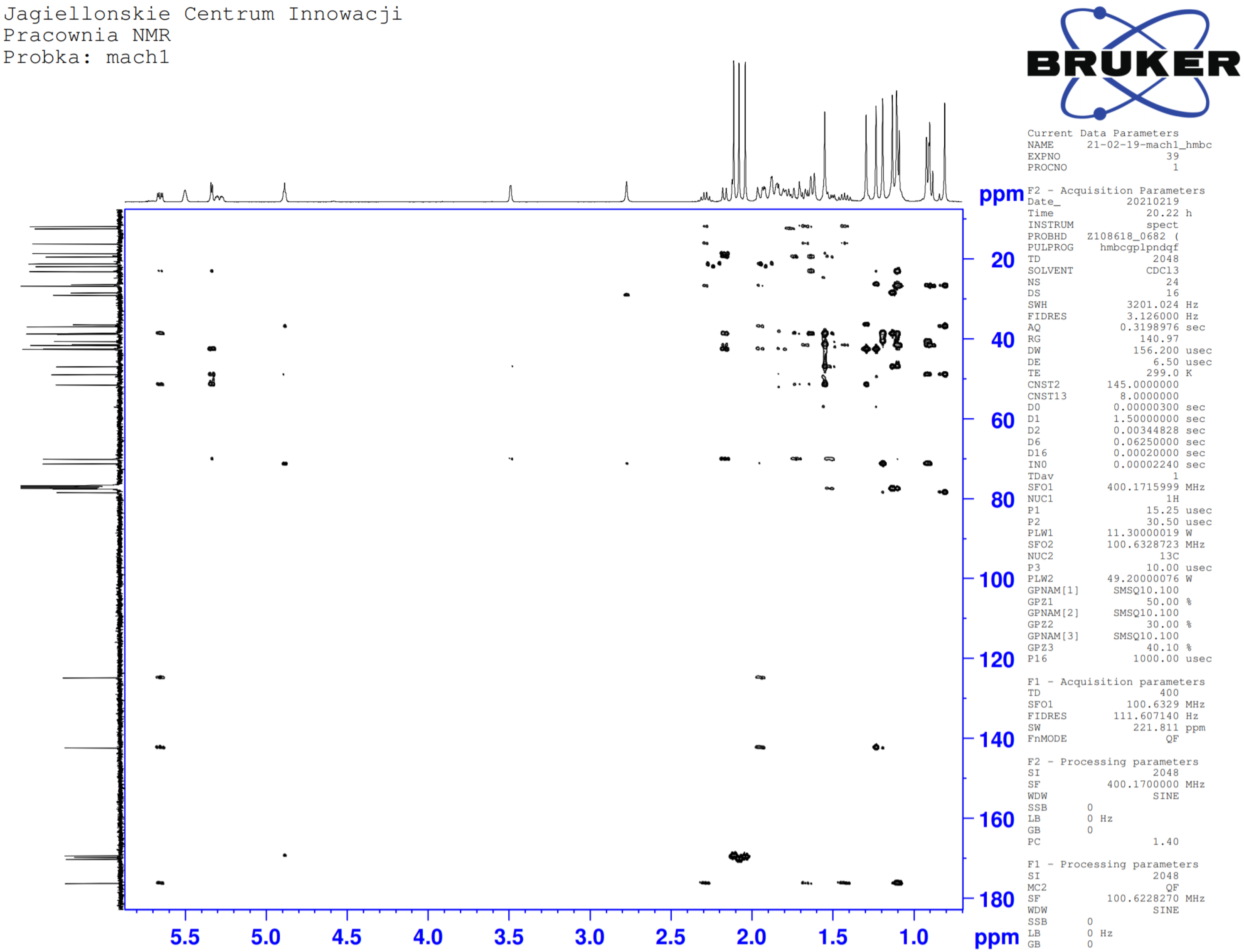

Figure S7. HMBC spectrum of compound **1**.

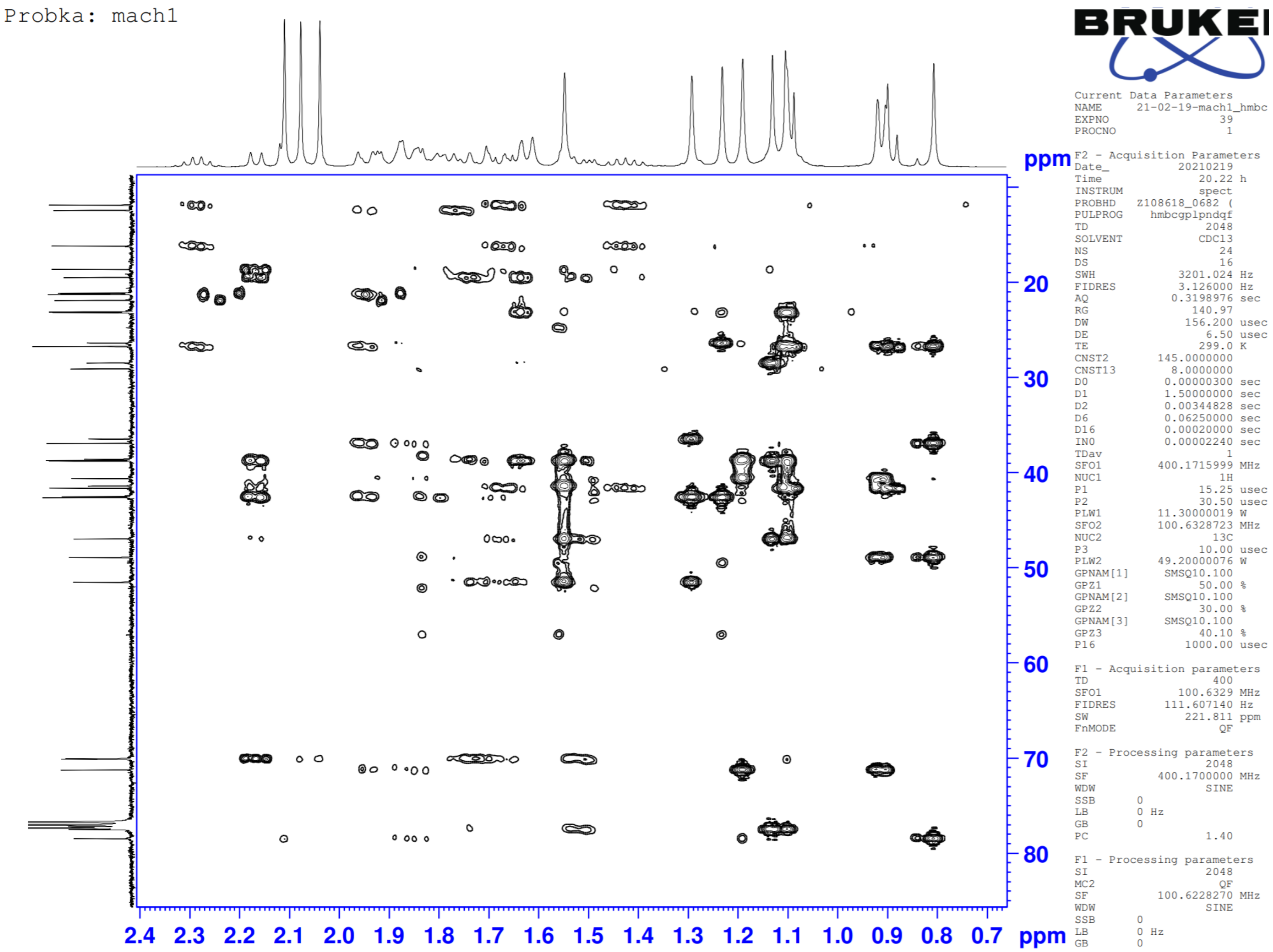

Figure S7a. Expansion of HMBC spectrum of compound **1**.

Single Mass Analysis
Tolerance = 3.0 mDa / DBE: min = -1.5, max = 100.0
Element prediction: Off
Number of isotope peaks used for i-FIT = 3
Monoisotopic Mass, Even Electron Ions
156 formula(e) evaluated with 2 results within limits (all results (up to 1000) for each mass)
Elements Used:
C: 0-100 H: 0-200 O: 0-9 Na: 0-1

Mass	Calc. Mass	mDa	PPM	DBE	Formula	i-FIT	i-FIT Norm	Fit Conf %	C	H	O	Na
655.3824	655.3822	0.2	0.3	8.5	C36 H56 O9 Na	589.3	0.013	98.66	36	56	9	1
	655.3846	-2.2	-3.4	11.5	C38 H55 O9	593.6	4.315	1.34	38	55	9	

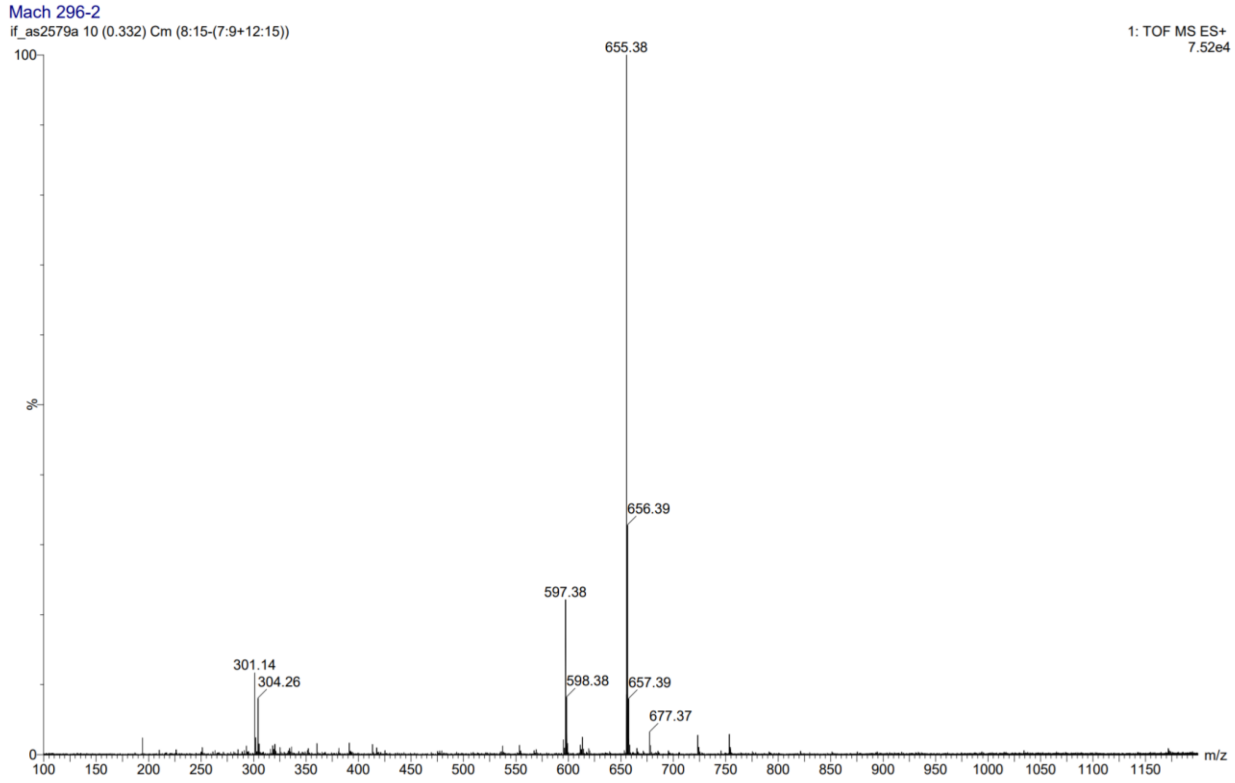

Figure S8. HR ESIMS spectrum of compound **2**.

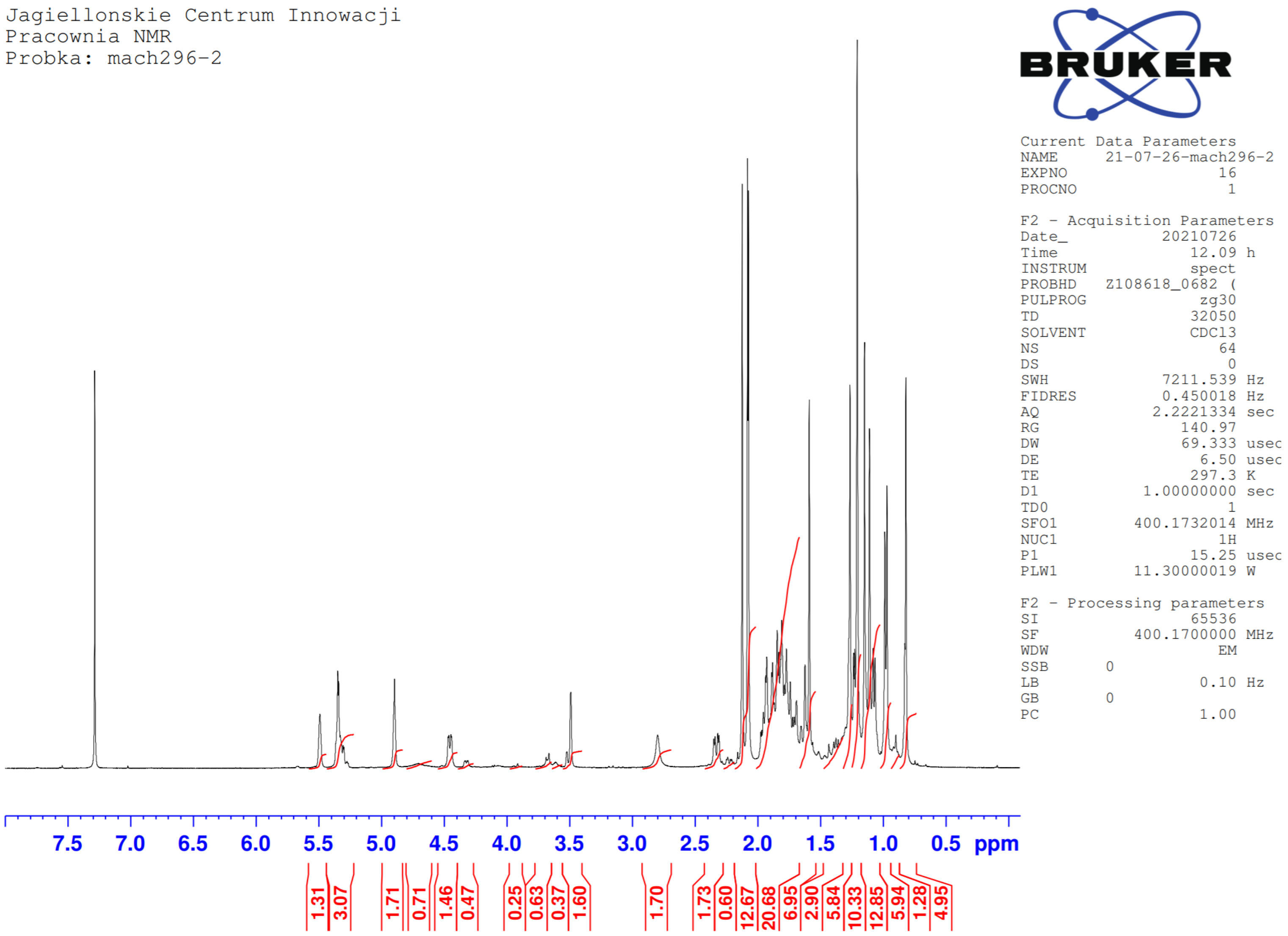

Figure S9. ^{1}H NMR spectrum of compound **2**.

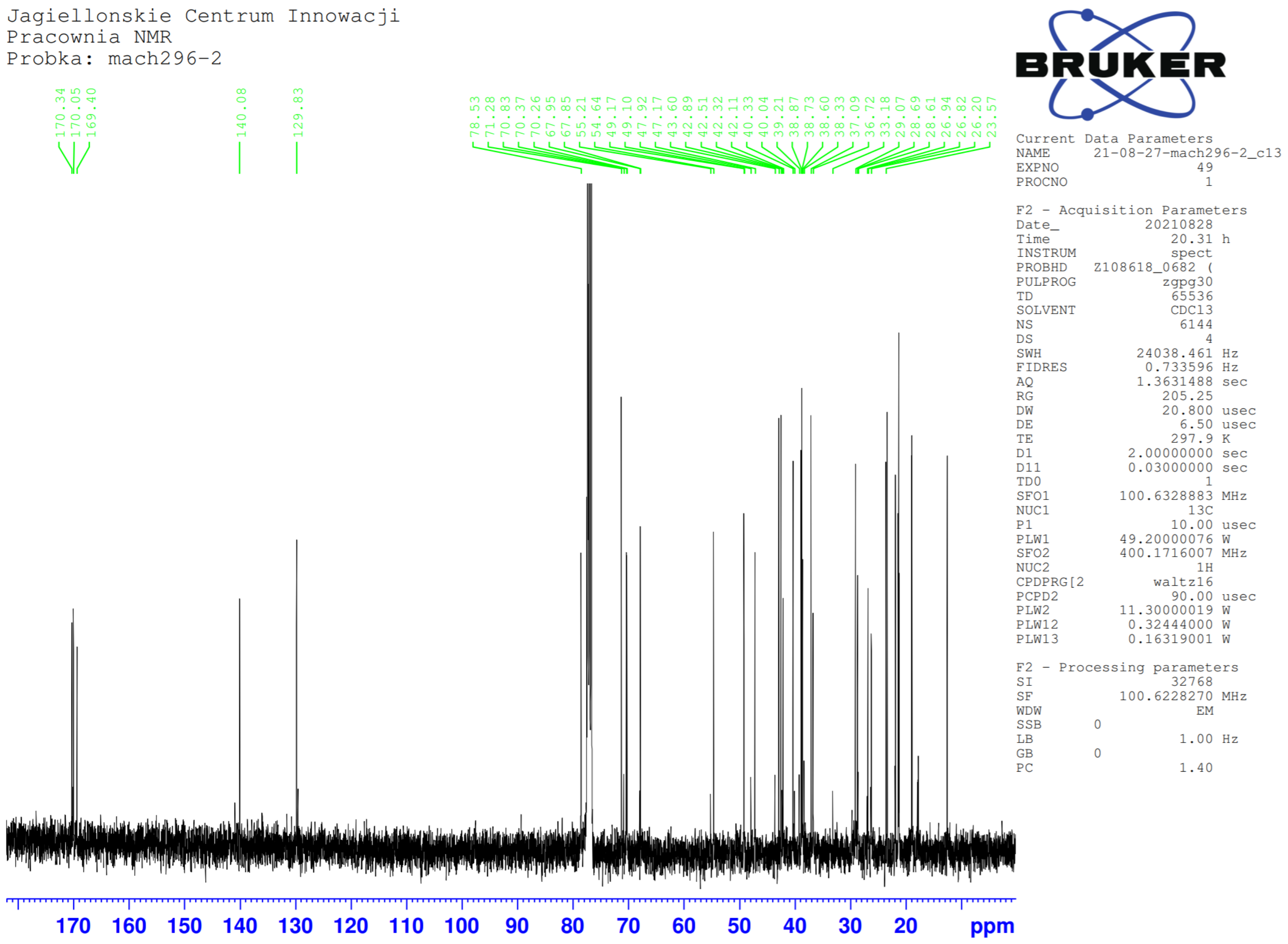

Figure S10. ^{13}C NMR spectrum of compound **2**.

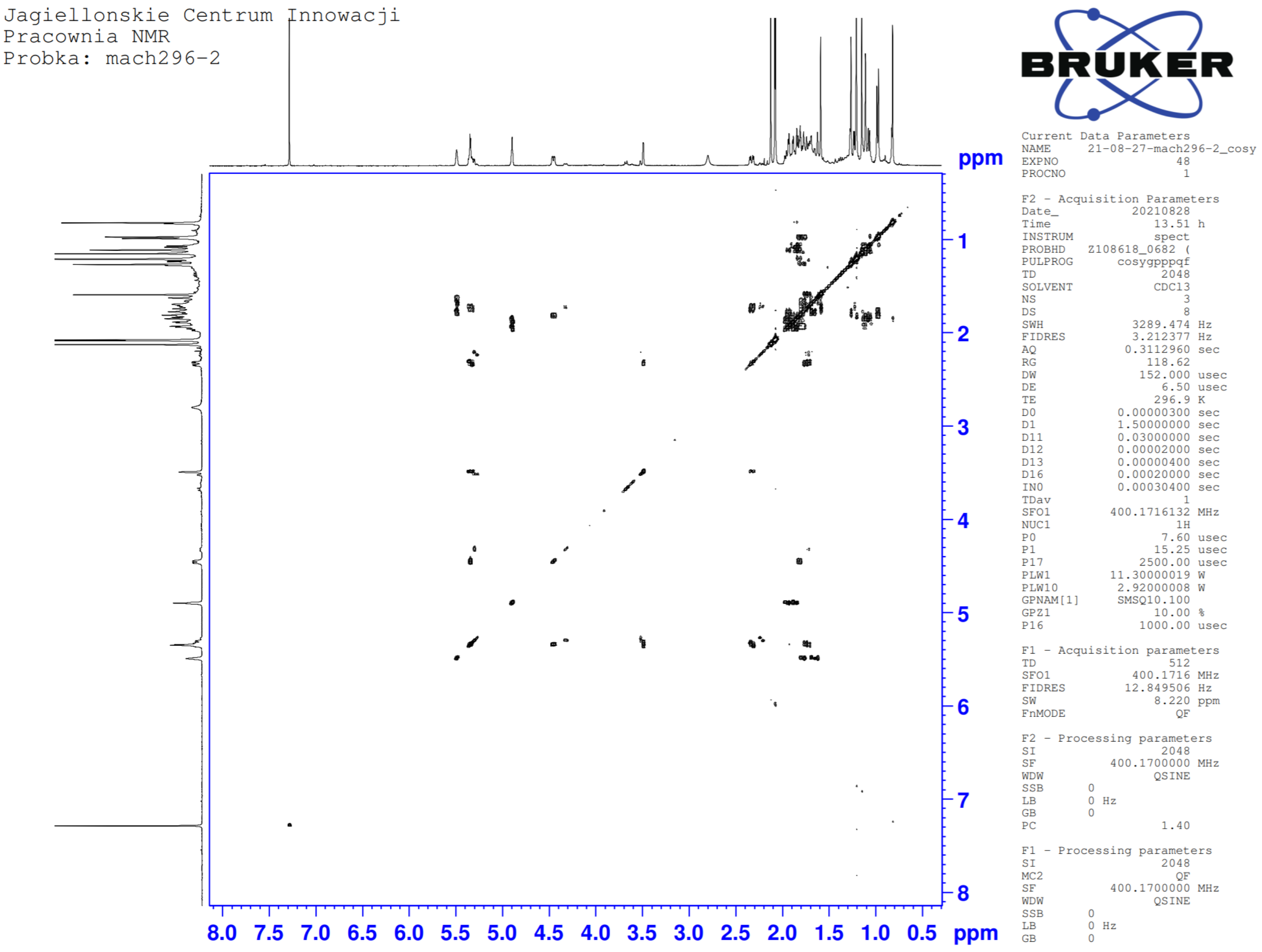

Figure S11. ^{1}H-^{1}H COSY spectrum of compound **2**.

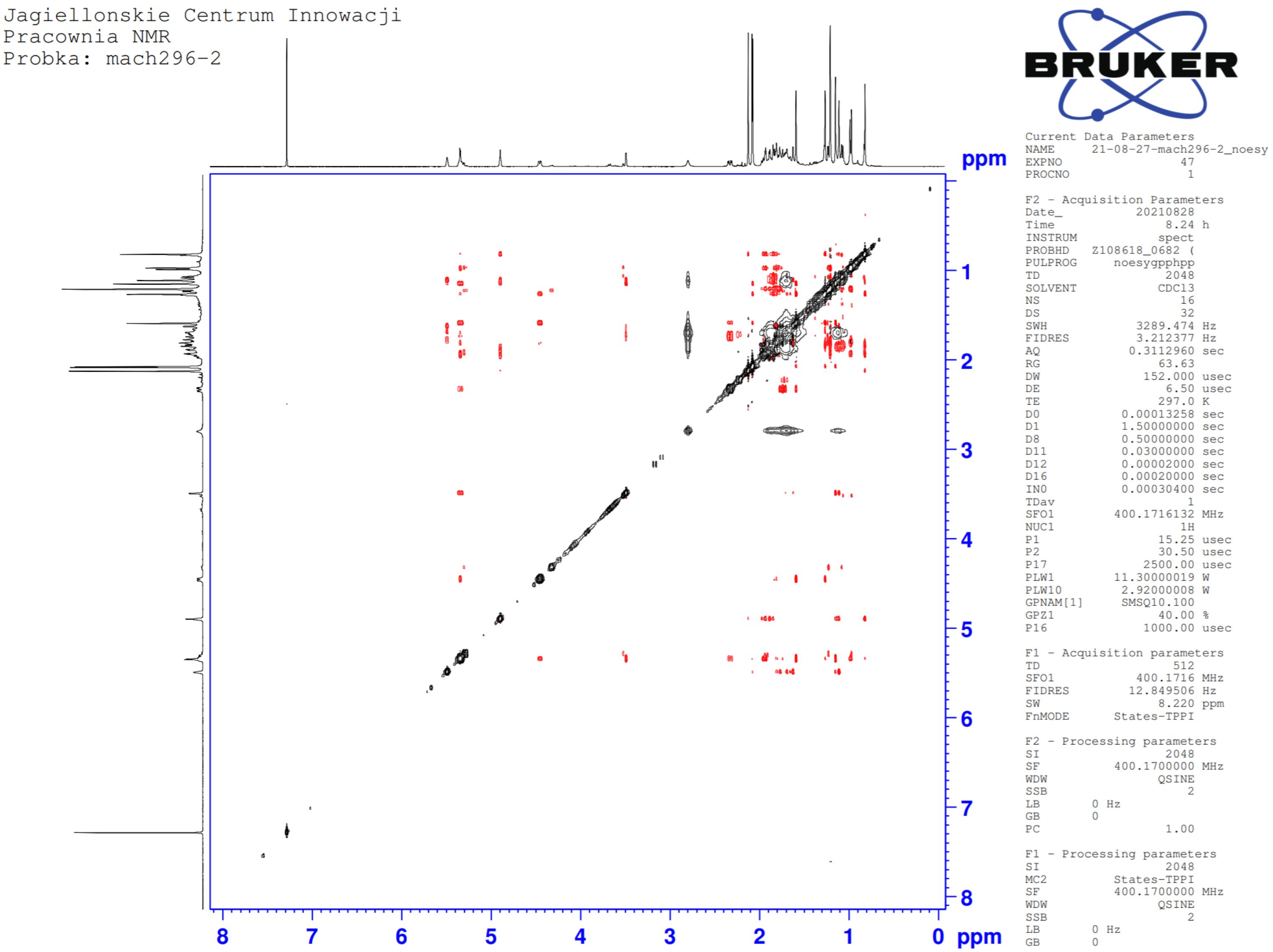

Figure S12. NOESY spectrum of compound **2**.

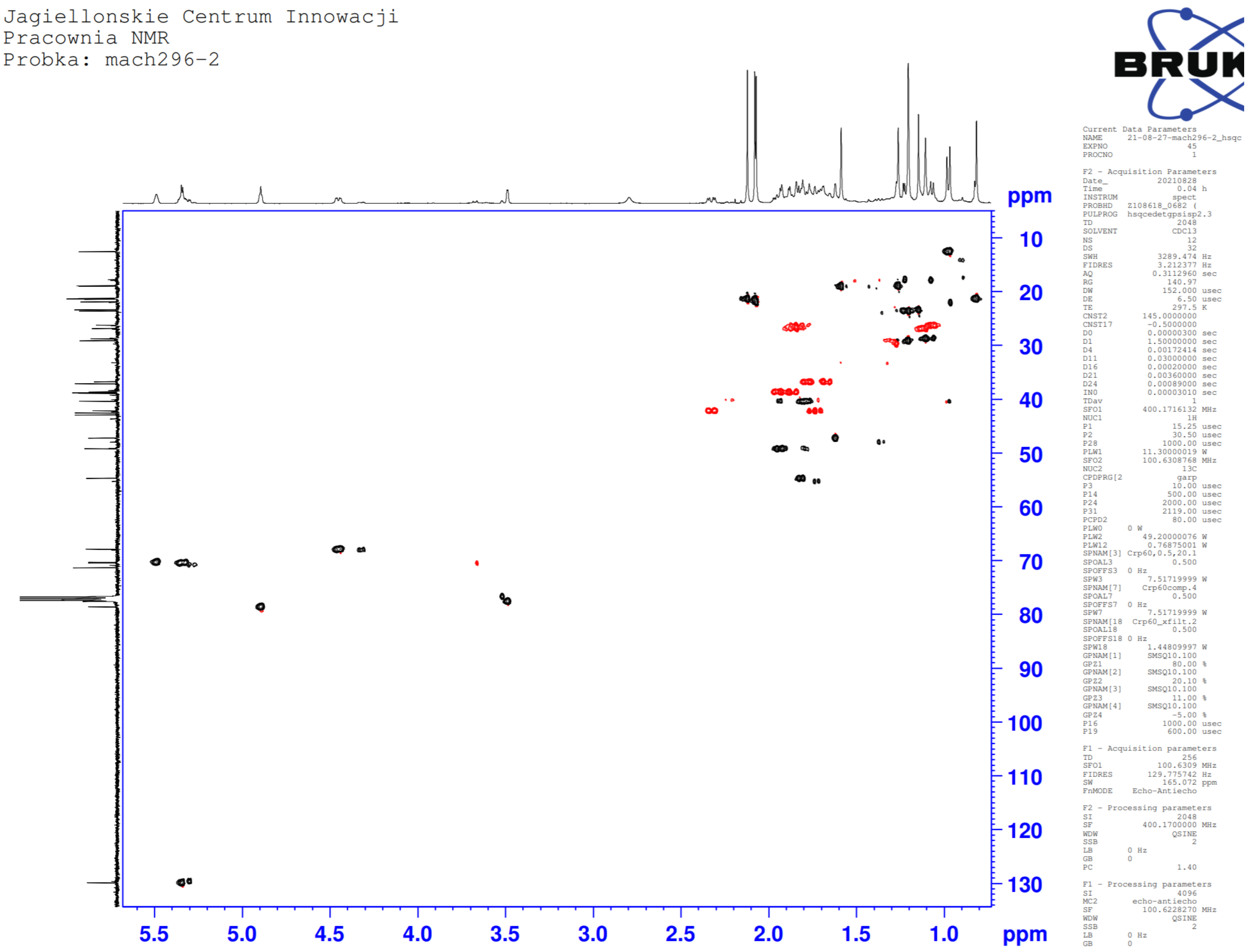

Figure S13. HSQC spectrum of compound **2**.

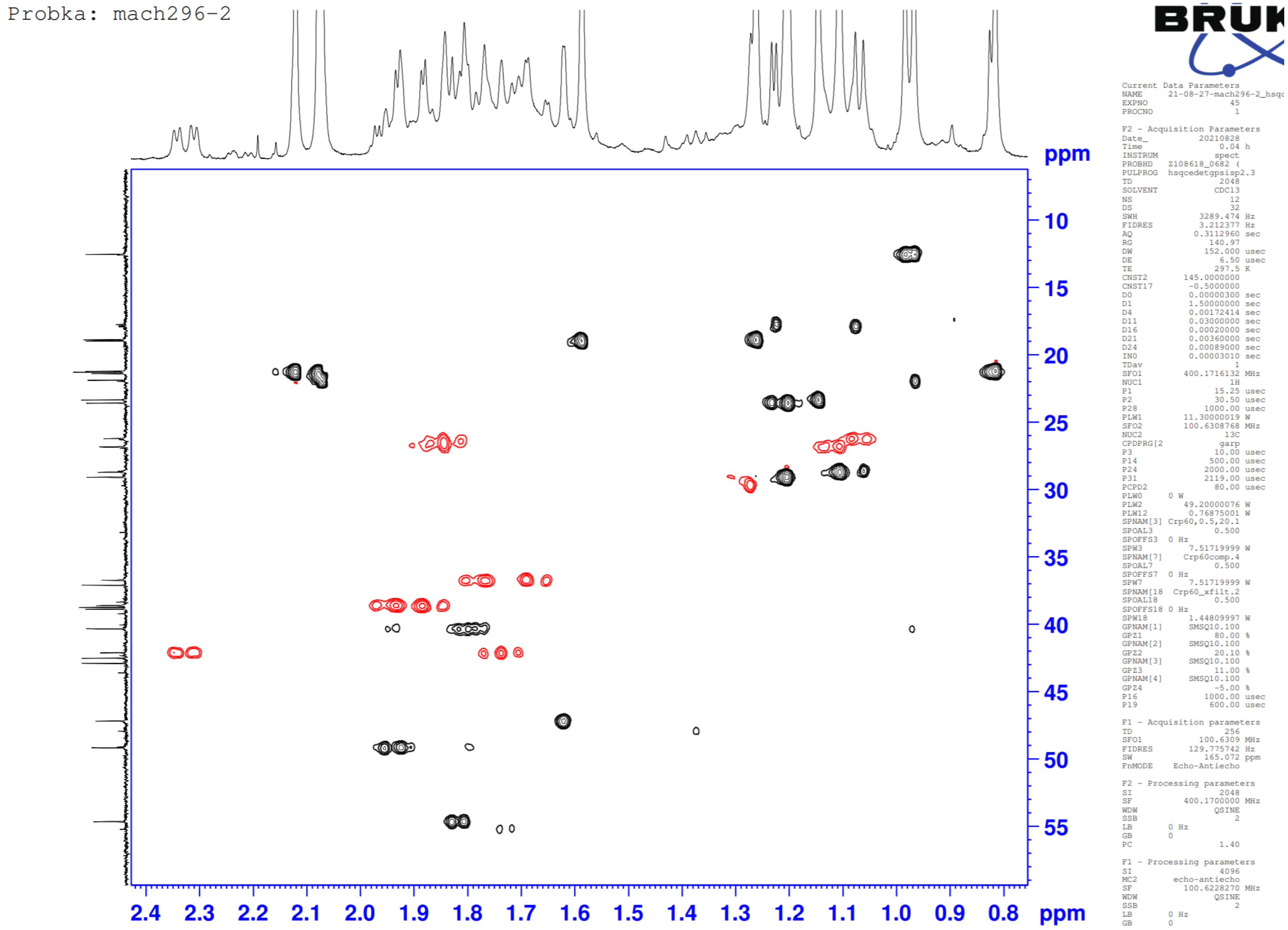

Figure S13a. Expansion of HSQC spectrum of compound **2**.

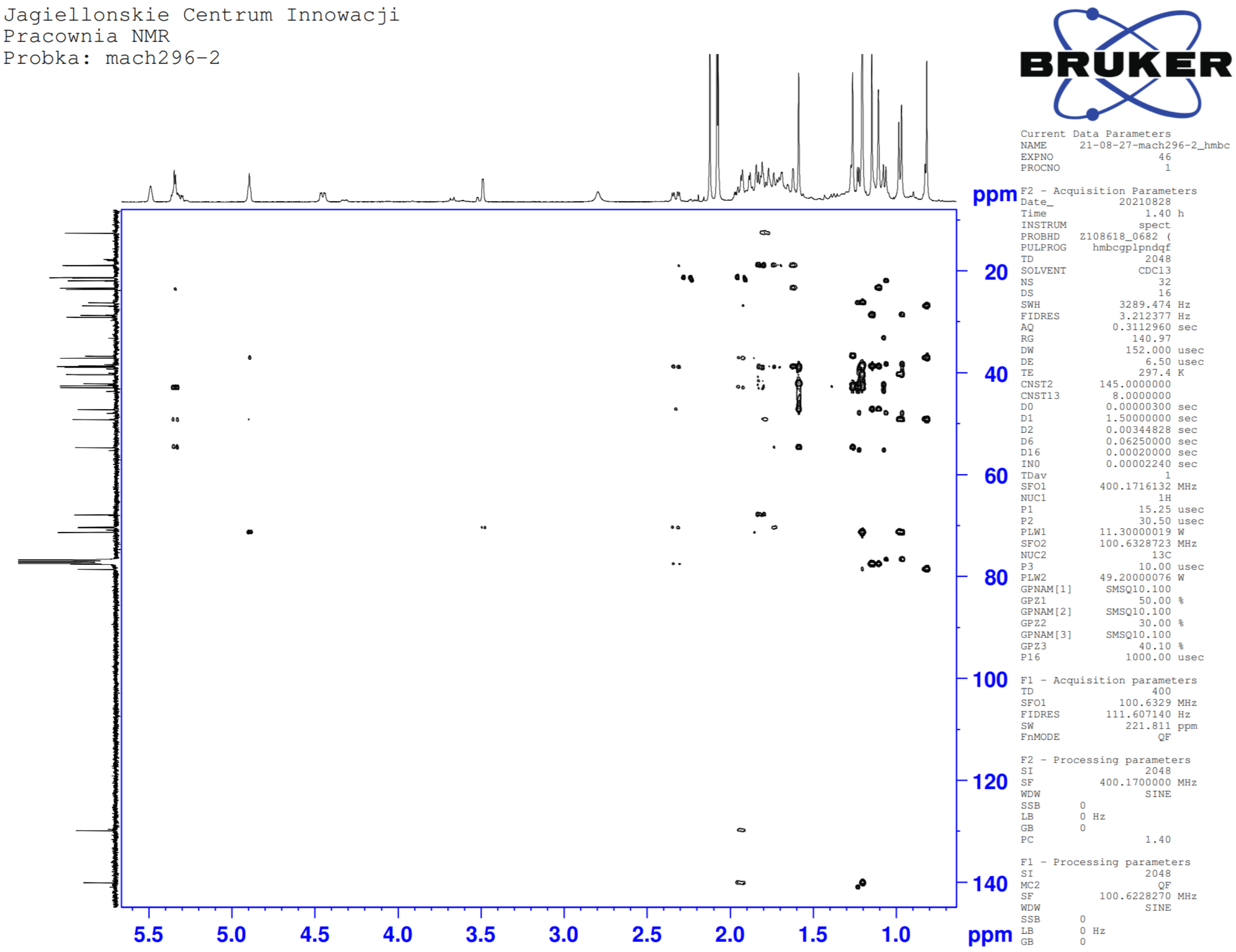

Figure S14. HMBC spectrum of compound **2**.

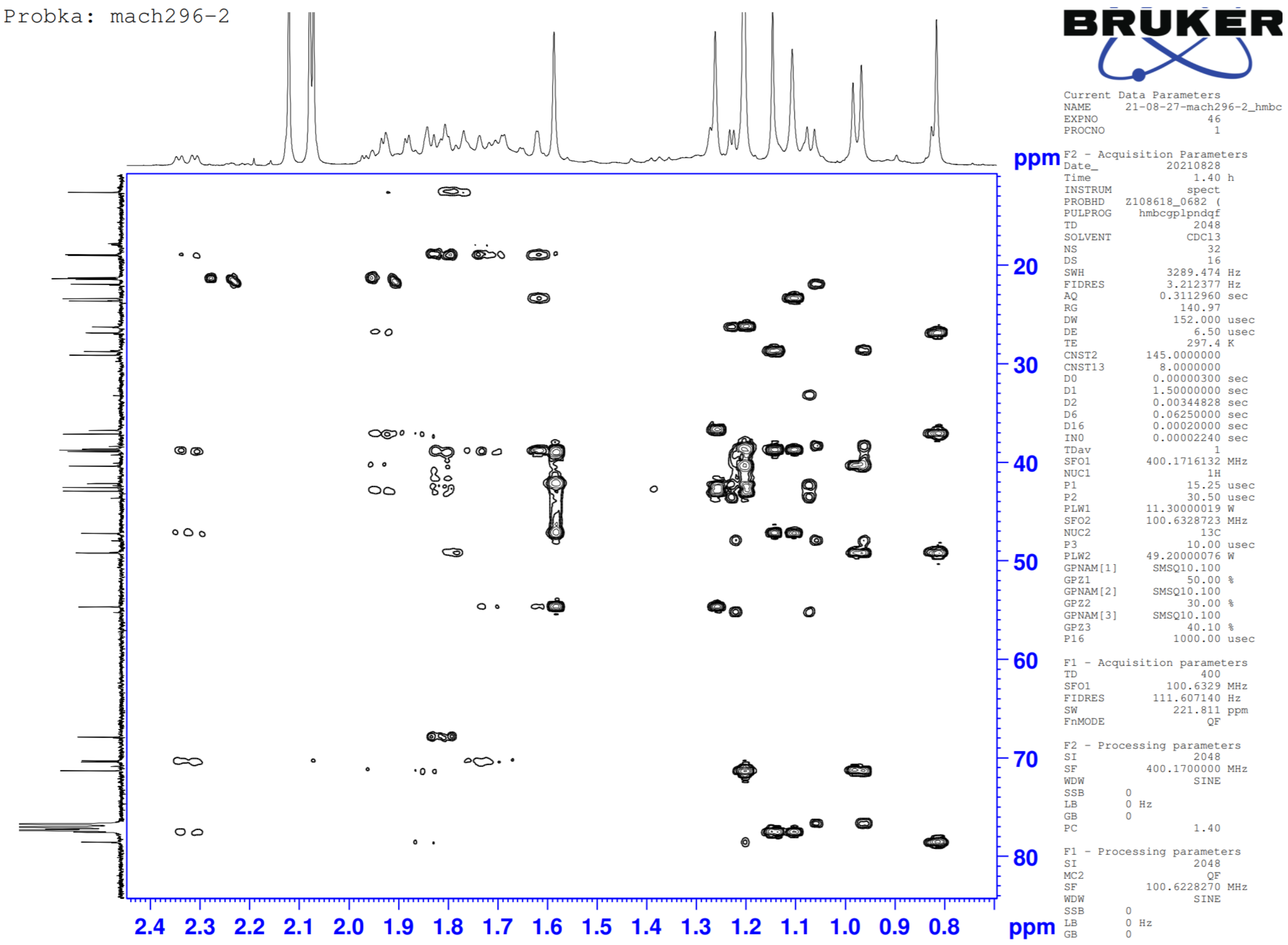

Figure S14a. Expansion of HMBC spectrum of compound **2**.

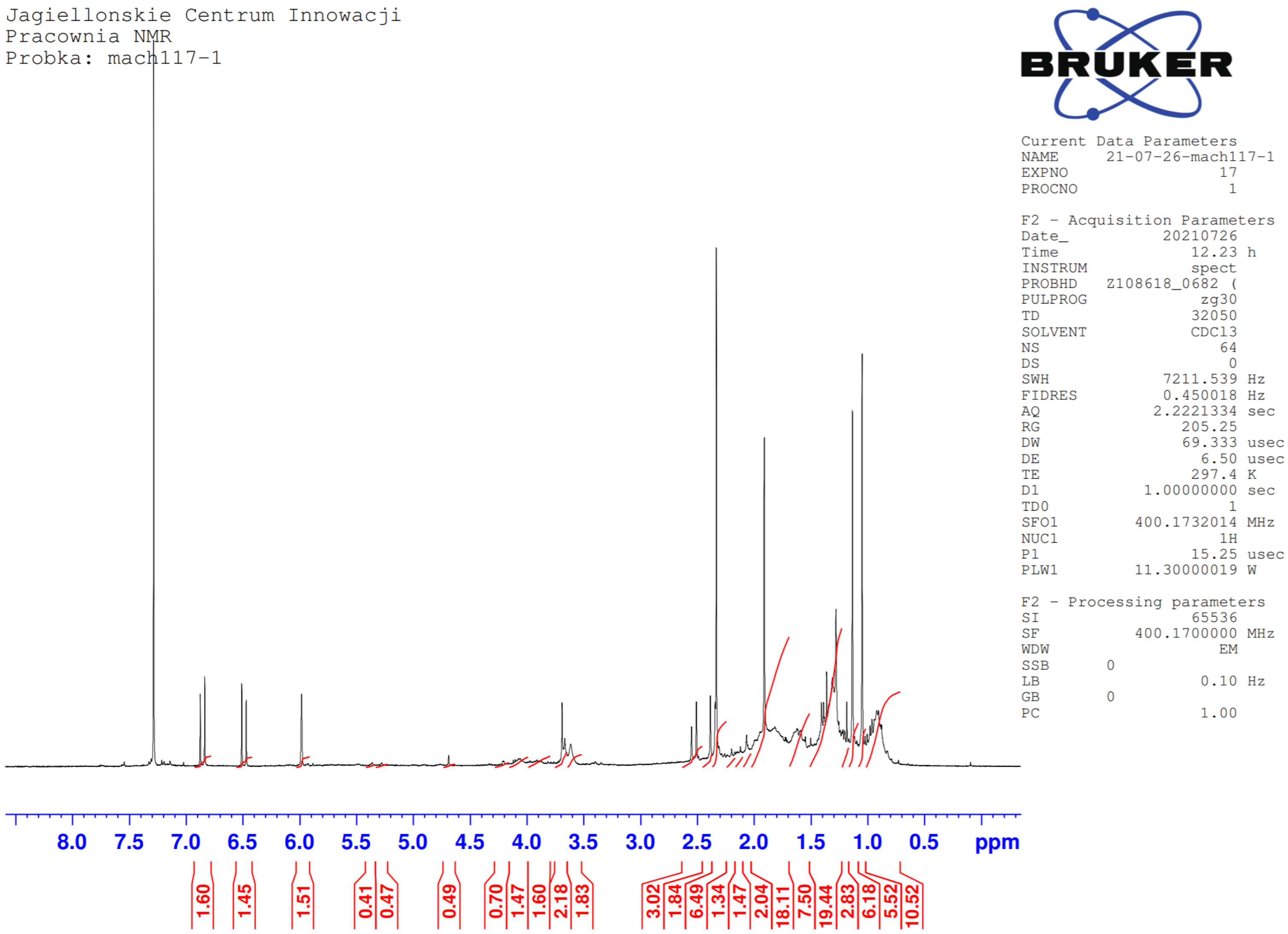

Figure S15. ^{1}H NMR spectrum of compound **3**.

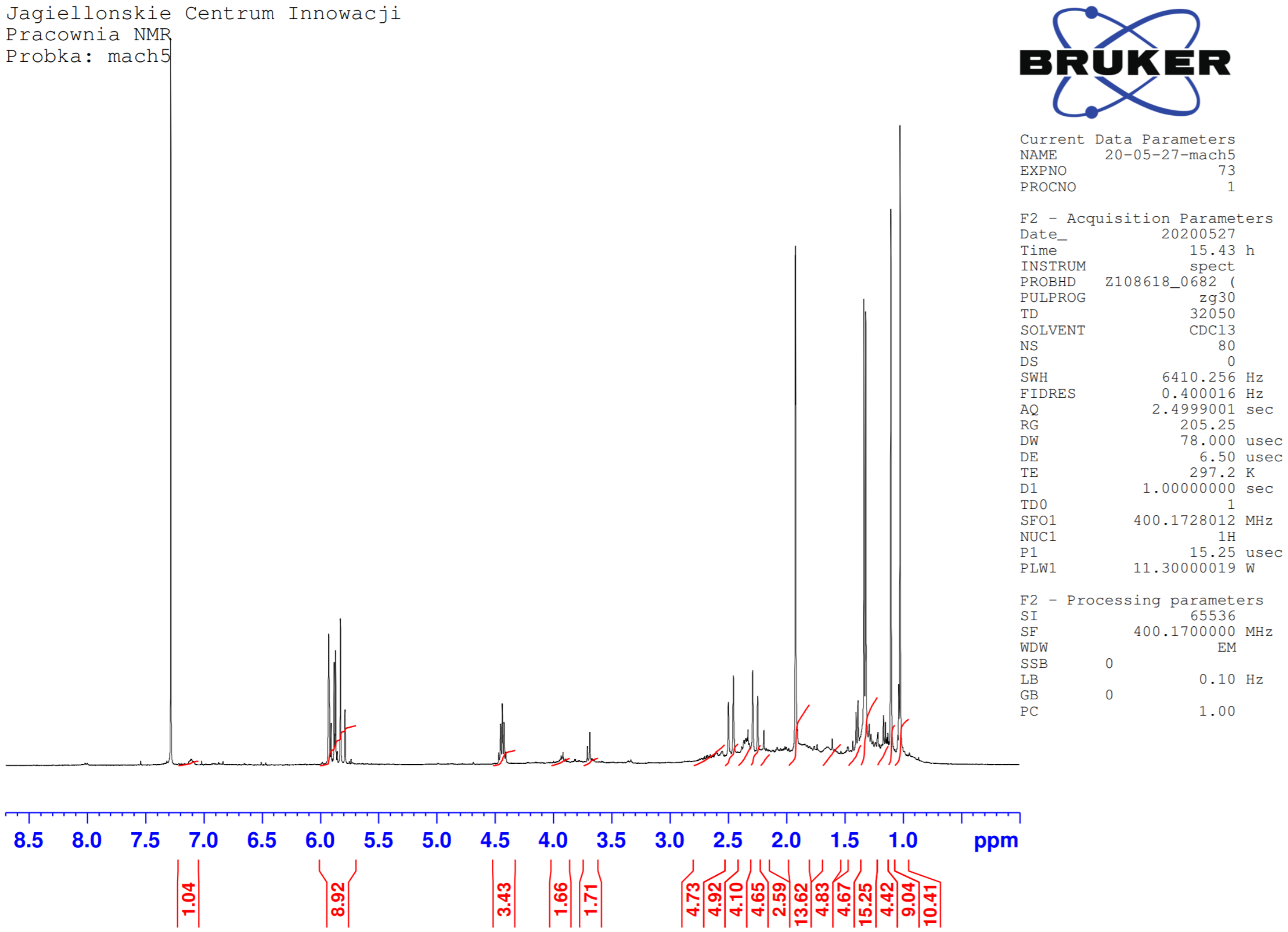

Figure S16. ^{1}H NMR spectrum of compound **4**.

Table S1. Viability of human normal and cancer cell lines treated for 24 h with 5-100 μg/mL of **1** or **2** (values are means of three measurements ± SD).

**Compound; dose**	**Cell viability [%] ± SD**							
	**Prostate normal and cancer cells**			**Keratinocytes and melanoma cells**			**Colon cancer**	
	**PNT-2**	**DU145**	**PC3**	**HaCaT**	**A375**	**HTB140**	**HT29**	**Caco-2**
**1**; 5 μg/mL	96.61 ± 0.53	93.19 ± 0.93	96.16 ± 1.04	96.52 ± 0.80	97.23 ± 0.57	96.15 ± 1.12	97.04 ± 0.42	95.55 ± 0.74
**1**; 10 μg/mL	96.77 ± 0.60	83.12 ± 1.96	90.71 ± 0.93	94.87 ± 1.00	96.44 ± 0.92	93.82 ± 0.73	95.49 ± 0.82	88.71 ± 0.93
**1**; 20 μg/mL	94.59 ± 0.90	70.39 ± 1.69	83.88 ± 1.67	88.37 ± 0.47	92.75 ± 1.11	87.08 ± 2.09	90.94 ± 1.37	78.46 ± 1.94
**1**; 30 μg/mL	91.05 ± 1.07	57.25 ± 2.20	74.81 ± 1.17	81.25 ± 1.77	87.25 ± 2.17	78.92 ± 2.08	81.48 ± 1.89	65.89 ± 1.21
**1**; 40 μg/mL	83.02 ± 1.41	41.26 ± 1.91	62.58 ± 1.36	73.95 ± 1.72	79.75 ± 2.02	68.39 ± 2.17	72.49 ± 1.94	51.13 ± 2.40
**1**; 50 μg/mL	70.73 ± 2.58	27.22 ± 2.28	45.67 ± 1.85	65.66 ± 1.22	70.18 ±1.39	59.51 ± 1.40	64.78 ± 1.69	35.78 ± 1.32
**1**; 100 μg/mL	59.02 ± 2.20	9.87 ± 1.59	28.14 ± 2.31	56.82 ± 2.20	62.56 ± 2.03	46.59 ± 2.09	55.21 ± 1.73	22.25 ± 1.53
**2**; 5 μg/mL	97.49 ± 0.79	97.30 ± 0.27	96.56 ± 1.35	96.84 ± 0.60	97.28 ± 0.33	97.48 ± 0.79	95.76 ± 0.69	94.66 ± 1.42
**2**; 10 μg/mL	96,75 ± 0.56	94.54 ± 0.58	94.14 ± 1.24	94.53 ± 1.55	95.28 ± 0.90	96. 66 ± 0.68	94.55 ± 0.84	88.42 ± 0.92
**2**; 20 μg/mL	96.30 ± 1.04	90.07 ± 0.71	87.92 ± 1.54	90.60 ± 0.66	91.07 ± 1.39	96.40 ± 0.83	90.05 ± 0.74	81.97 ± 1.69
**2**; 30 μg/mL	95.64 ± 0.65	83.15 ± 1.24	78.96 ± 1.10	82.83 ± 1.23	87.27 ± 1.50	94.29 ± 1.45	81.65 ± 1.56	74.70 ± 1.19
**2**; 40 μg/mL	95.37 ± 0.75	77.13 ± 1.63	70.32 ± 1.60	75.68 ± 1.76	82.63 ± 1.36	93.72 ± 1.11	74.77 ± 1.13	65.80 ± 1.25
**2**; 50 μg/mL	94.56 ± 0.32	68.62 ± 1.78	58.98 ± 1.81	61.79 ± 1.55	76.22 ± 1.78	91.95 ± 1.64	67.15 ± 1.25	61.07 ± 2.37
**2**; 100 μg/mL	93.56 ± 0.50	57.30 ± 1.90	47.10 ± 2.64	54.26 ± 1.78	67.56 ± 1.48	89.49 ± 1.40	56.91 ± 1.85	49.61 ± 1.49
